# Compound Analysis and Mechanistic Exploration of Shen‐Yan‐Fang‐Shuai Formula in Diabetic Kidney Disease: An UHPLC‐MS/MS and Bioinformatics Study

**DOI:** 10.1155/jdr/7530512

**Published:** 2026-04-24

**Authors:** Yi Kang, Qian Jin, Mengqi Zhou, Huijuan Zheng, Danwen Li, Xuezhe Wang, Jingwei Zhou, Jie Lv, Yaoxian Wang

**Affiliations:** ^1^ Dongzhimen Hospital, Beijing University of Chinese Medicine, Beijing, China, bucm.edu.cn; ^2^ Graduate School of Beijing University of Chinese Medicine, Beijing, China, bucm.edu.cn; ^3^ Department of Traditional Chinese Medicine, Beijing Puren Hospital, Beijing, China

**Keywords:** diabetic kidney disease, machine learning, molecular dynamics simulation, network pharmacology, shen-yan-fang-shuai formula, UHPLC-MS/MS

## Abstract

**Background:**

Diabetic kidney disease (DKD) is significantly impacting both quality of life and survival rates. The Shen‐Yan‐Fang‐Shuai (SYFS) formula is a traditional Chinese medicine (TCM) compound widely used in the clinical treatment of DKD with proven efficacy, though its potential mechanism of action remains unclear. This study attempts to elucidate the therapeutic efficacy, mechanisms of action, and active compounds of the SYFS formula in the treatment of DKD.

**Materials and Methods:**

The components of SYFS formula were identified by UHPLC‐MS/MS. Differentially expressed genes (DEGs) and key module genes were selected based on the GEO database to obtain intersection targets. Protein–protein interaction (PPI) network and component‐target network were constructed. Machine learning (ML) was employed to screen for hub genes, which were validated through nomogram, immune infiltration analysis, molecular docking and molecular dynamics (MD) simulation. Subsequently, our findings were validated through a combination of transcriptomic sequencing of renal tissue from animal models and real‐time quantitative PCR (qPCR) analyses performed on both the animal tissues and HK‐2 cells.

**Results:**

154 chemical components and 994 targets were identified in the SYFS formula. Intersection with DEGs and WGCNA module genes identified 39 potential targets. Five hub genes (MMP3, MMP12, PTGES, SST, and DUSP1) were selected through ML and used to construct a nomogram. Multiple immune cell infiltration levels were significantly elevated in DKD, with hub genes showing correlations with specific immune cell types. Molecular docking and MD simulation validated the binding capacity between components of the SYFS formula and key targets. In addition, it has been further verified in animal experiments and cell experiments.

**Conclusions:**

The core components of the SYFS formula, including naringenin chalcone, palmatine, oleanonic acid, *β*‐elemonic acid, and Naringenin, likely exert their effects through the MMP3, MMP12, PTGES, SST, and DUSP1 targets. This research offers empirical support for the application of the SYFS formula in DKD, establishing a crucial groundwork for subsequent clinical investigations.

## 1. Introduction

Diabetic kidney disease (DKD) involves a complex and heterogeneous pathological process. Throughout the disease course, the glomeruli, renal tubules, renal interstitium, and vasculature are all affected. Recent data indicate that from 1990 to 2021, global disability‐adjusted life years for DKD‐T1DM and DKD‐T2DM increased by 74.0% and 173.6%, respectively, with continued growth projected through 2030, imposing substantial health and economic burdens on patients, families, and society [1]. Although novel hypoglycemic agents (such as SGLT2 inhibitors and GLP‐1 receptor agonists) have shown efficacy in delaying DKD progression in recent years, some DKD patients still face the risk of renal failure [2]. More critically, the pathological mechanisms of DKD involve complex interactions among multiple processes, including hemodynamics, glucose and lipid metabolism, oxidative stress, immunity, inflammation, and fibrosis [3, 4], making it difficult for single‐target drugs to comprehensively block this complex pathological network.

Traditional Chinese medicine (TCM) has accumulated over a 1000 years of practical experience in treating chronic kidney disease, with traditional Chinese herbs delaying DKD progression through multiple targets and pathways [5]. Shen‐Yan‐Fang‐Shuai formula (SYFS formula) is a clinically verified prescription for treating DKD. It has demonstrated effectiveness in reducing proteinuria in DKD patients and improving renal function [6]. Furthermore, it delays the onset of dialysis for patients with stage 5 chronic kidney disease (CKD5) during the nondialysis period and reduces the occurrence of dialysis events in CKD5 patients [7]. Empirical research has demonstrated that SYFS formula intervention significantly reduced proteinuria in DKD rat models, inhibited the MAPK pathway to decrease renal tissue inflammation, and improved renal pathological damage [8]. It may also exert a renal protective effect by inhibiting inflammatory responses and extracellular matrix (ECM) accumulation mediated by the TNF‐*α*/NF‐*κ*B p65 signaling pathway [9]. At the same time, SYFS formula treatment improved intestinal barrier function in high‐fat diet (HFD)‐fed mice, reduced metabolic endotoxemia, systemic inflammation, and insulin resistance [10]. However, its specific targets and molecular network mechanisms have not been systematically elucidated.

Network pharmacology, through constructing multilevel network models of disease‐drug‐target interactions, systematically reveals the essence of Chinese medicine compounds’ “multi‐component synergy and multi‐pathway integration,” aligning with TCM’s holistic concept and syndrome differentiation theory [11–13]. Bioinformatics provides powerful tools for analyzing the complex action networks, and this interdisciplinary research approach enables us to comprehensively understand the complex interaction network at a systems level [14]. This study employs network pharmacology and bioinformatics approaches to investigate the molecular mechanisms of SYFS formula in DKD treatment.

**Figure 1:**
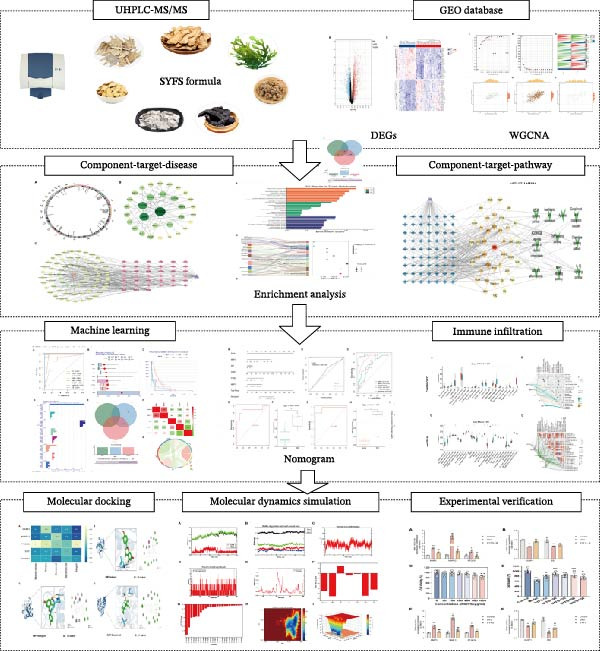
Research flowchart.

We systematically identified components and targets of SYFS formula using UHPLC‐MS/MS nontargeted metabolomics technology and integrated them with gene expression profiles from GEO database [15] to obtain drug‐disease intersection targets. Unlike most network pharmacology studies that remain at the predictive level, we deeply integrated machine learning (ML) algorithms to precisely screen core targets from vast network data. In addition, we combined molecular dynamics simulations to evaluate the binding stability. Finally, the predictive results were validated through animal and cell experiments. This formed a complete “computational prediction‐experimental validation” feedback loop (Figure 1). This study demonstrates the potential mechanisms of SYFS formula in treating DKD at molecular and pathway levels, providing new evidence for TCM treatment of DKD.

## 2. Methods

### 2.1. Preparation of SYFS Formula and UHPLC‐MS/MS Analysis

The SYFS formula contained the following medicinal plants (108 g in total: Huang qi (*Astragalus mongholicus* Bunge) 30 g, Dang gui (*Angelica sinensis* (Oliv.) Diels) 10 g, Hai Zao (*Phoenix dactylifera* L.) 10 g, Muli (Concha ostreae) 15 g, Bie Jia (Trionyx sinensis Wiegmann) 10 g, Shu Dihuang (*Rehmannia glutinosa* (Gaertn.) Libosch. ex DC.) 30 g, San qi (*Panax notoginseng* (Burkill) F.H.Chen 3 g (Table 1). All names are in accordance with the latest revised version of World Plants Online (https://www.worldfloraonline.org/).

**Table 1:** Drug composition of SYFS formula.

No.	Pinyin name	Pharmaceutical latin	Dose (g)
1	Huang qi	*Astragalus mongholicus*	30
2	Dang gui	*Angelica sinensis*	10
3	Hai Zao	*Phoenix dactylifera*	10
4	Mu li	*Concha ostreae*	15
5	Bie Jia	*Trionyx sinensis Wiegmann*	10
6	Shu Dihuang	*Rehmannia glutinosa*	30
7	Sanqi	*Panax notoginseng*	3

All seven herbs in the SYFS formula were purchased from Dongzhimen Hospital, Beijing University of Chinese Medicine, and authenticated by the hospital’s pharmacy department. Following the standard method stipulated in the Pharmacopoeia of the People’s Republic of China (Chinese Pharmacopoeia Commission, 2020), SYFS was prepared. Prior to decoction, each herb was soaked in water (1:10 w/v) for 30 min. Initially, Bie Jia (*Trionyx sinensis Wiegmann*) and Muli (*Concha ostreae*) were decocted for 30 min, followed by the addition of the remaining herbs and further decoction for 30 min, then filtered to obtain extract A. The residue was redissolved in water, decocted for another 30 min, and filtered to obtain extract B. Extracts A and B were combined, concentrated, transferred to freezing dishes, and frozen at −80°C for 24 h. Finally, the concentrated solution was lyophilized at −80°C, powdered, packaged, and stored at −80°C. UHPLC‐MS/MS analysis is detailed in Supporting Information 1.

### 2.2. Drug Targets of SYFS Formula

The SMILES codes for the ingredients in SYFS formula were obtained from the PubChem database (https://pubchem.ncbi.nlm.nih.gov/). The SMILES codes were uploaded to STITCH (http://stitch.embl.de/) and SwissTargetPrediction (http://www.swisstargetprediction.ch/) for analysis, where the SwissTargetPrediction database selected targets with a probability ≥ 0.1, and the STITCH database selected potential targets with medium confidence ≥0.4. After merging and deduplication, the drug targets for SYFS formula were obtained.

### 2.3. Differentially expressed genes (DEGs) of DKD

DKD expression datasets were downloaded from GEO database: GSE96804 and GSE30528 as training set, while GSE30529 and the Woroniecka Diabetes Glom dataset from Nephroseq (http://v5.nephroseq.org) as validation set (Table 2). The combat algorithm of Sangerbox online analysis platform (http://www.sangerbox.com/) [16] was used to batch remove the training set. DEGs (limma package [17]) selected based on the criteria: fold change (FC) > 1.2 and adj.*p* < 0.05 [18, 19]. Overview of the datasets are detailed in Table 2.

**Table 2:** Summary of the data sets utilized in this research.

Dataset	Database	Platform	Sample	Tissue origin
GSE96804	GEO	GPL17586	41 cases of DKD and 20 cases of controls	Glomeruli
GSE30528	GEO	GPL571	9 cases of DKD and 13 cases of controls	Glomeruli
GSE30529	GEO	GPL571	10 cases of DKD and 12 cases of controls	Tubuli
Woroniecka Diabetes Glom	Nephroseq	/	9 cases of DKD and 13 cases of controls	Glomeruli

### 2.4. Weighted Gene Correlation Network Analysis

The training set utilized the “WGCNA” package [20] to construct gene coexpression network. The median absolute deviation (MAD) screening method was employed to eliminate the 50% of genes with the lowest expression variability. The adjacency matrix was optimized through a soft threshold parameter (*β*) and subsequently transformed linearly to generate a topological overlap matrix (TOM). Based on the dissimilarity matrix of the TOM, gene expression profiles were modularly grouped using average linkage hierarchical clustering (with a minimum module gene count of 50). After calculating module eigenvector values, dynamic cutting algorithms were applied to determine the cutting height of the dendrogram, ultimately merging similarity modules. Finally, key modules were identified, and a topologically visualized gene coexpression network was constructed.

### 2.5. Component‐Target Network

The intersection of drug target, DEGs and module gene was taken as the potential target of SYFS formula therapy for DKD. These potential targets were imported into the STRING database (https://string-db.org/) [21], with confidence settings ≥0.9 and species specified as “*Homo sapiens*”, to establish protein–protein interaction (PPI) network. Cytoscape 3.9.1 was employed to visualize the relationships between components and targets.

### 2.6. Enrichment Analysis

The clusterProfiler package [22] was used to conduct gene ontology (GO) and kyoto encyclopedia of genes and genomes (KEGG) enrichment analysis. The top 10 pathways ranked by significance in the KEGG enrichment analysis were further selected, and Cytoscape 3.9.1 was utilized to construct component‐target‐pathway network, enabling in‐depth exploration of the principal components, targets, and pathways through which SYFS formula exerts its therapeutic effects in DKD.

### 2.7. ML to Identify Hub Genes

The R packages caret, randomForest, kernlab, and xgboost were used to construct 8 ML models, including random forest (RF), support vector machine (SVM), generalized linear model (GLM), gradient boosting machine (GBM), k‐nearest neighbors (KNN), neural network (NNET), LASSO, and decision tree (DT). The DALEX package was used to analyze and visualize the residual distribution and gene importance, thereby identifying the most effective ML model and key feature genes.

### 2.8. Nomogram Model

The rms and rmsa packages were used to construct Nomogram model. Additionally, a calibration curve was used to assess the consistency between the predicted probabilities and actual outcomes. To validate their discriminative ability, ROC analysis was performed to assess their predictive accuracy in distinguishing between disease and normal states. The area under the curve (AUC) value quantifies the ability of each gene to classify categories, with a higher AUC indicating better performance. The model was validated using the validation set data, and the correlation between hub genes and clinical markers, such as serum creatinine (Scr) and glomerular filtration rate (GFR), was analyzed.

### 2.9. Immune Infiltration Analysis

The CIBERSORT algorithm was used to analyze the immune infiltration levels (PERM = 100 and *p*‐value threshold of 0.05). Single‐sample Gene Set Enrichment Analysis (ssGSEA) is an extension of the Gene Set Enrichment Analysis (GSEA) algorithm. ssGSEA computes an enrichment score separately for each sample and for each gene set, and it can be used to assess differences in immune infiltration at the level of individual samples. This procedure is implemented using the GSVA package. The ssGSEA algorithm converts a normalized gene expression matrix into an enrichment‐score matrix; the resulting ssGSEA enrichment scores represent the relative abundance of each immune cell population in each sample. The corrplot package was used to generate correlation heatmaps to visualize the relationships between hub genes and the immune cells.

### 2.10. Consensus Clustering

Based on hub genes, unsupervised clustering analysis was performed on 50 DKD samples using the ConsensusClusterPlus package [23]. The similarity among the samples was determined via Euclidean distance, which was subsequently subjected to K‐means clustering. Thereafter, 500 iterations were conducted with a resampling proportion of 0.8. The optimal number of clusters was determined through comprehensive evaluation of the cumulative distribution function (CDF) curves, consensus matrix, and consistency clustering scores. Based on immune infiltration results, the levels of immune infiltration under different clustering patterns of DKD were further analyzed. GSVA is an unsupervised gene set enrichment analysis method, and the GSVA package was employed for GSVA enrichment analysis.

### 2.11. Molecular Docking and Molecular Dynamics (MD) Simulation

The 2D structure of the small molecule ligand was obtained from the PubChem database, and its 3D structure was generated using ChemOffice software. A protein target with a high‐resolution crystal structure was selected from the RCSB PDB database to serve as the receptor for molecular docking. The protein was processed using PyMOL software to remove water molecules, phosphate groups, and other modifications, and the processed structure was saved as a PDB file. Molecular docking was performed using AutoDock Vina 1.5.6 software, and the docking results were visualized using Discovery Studio 2019 software. To gain a deeper understanding of the stability of protein‐ligand interactions, a 100 ns MD simulation was performed on the complex using GROMACS 2025.2 package. Detailed steps can be found in Supporting Information 1.

### 2.12. Animal Experiment Verification

48 SPF‐grade male C57BL/6J mice (weight 20 ± 2 g, 6–8 weeks) were purchased from Beijing Vital River Laboratory Animal Technology Co., Ltd. (Production License No.: SCXK (Beijing)‐20210006). This study was approved by the Animal Ethics Committee of Beijing University of Chinese Medicine (BUCM‐2023120104‐4282). Forty‐eight mice were randomly divided into Control group (*n* = 12) and Model group (*n* = 36) using a random number table method. The DKD mouse model was established based on the referenced methodology [24]. Details on animal feeding conditions and experiment procedures in Supporting Information 1. The mice were then fed an HFD for 20 weeks. After 20 weeks, the successfully modeled mice were randomly divided into DKD group (*n* = 12), SYFS group (*n* = 12), and dapagliflozin (DA) group (*n* = 12). Based on the body surface area conversion between humans and mice, the equivalent dose for mice was calculated to be ~16.38 g/kg. The dose for the DA group was selected as 1.5 mg/kg/day, according to the referenced literature [25, 26]. The Control and DKD groups were administered an equal volume of physiological saline by gavage, once daily for 12 weeks. Blood was collected from the heart under anesthesia after 12 weeks. After complete renal dissection, the cortical tissue was separated into two parts: one part was fixed in 4% paraformaldehyde for 24 h for histopathological evaluation using HE/PAS/Masson staining, while the other part was snap‐frozen in liquid nitrogen. Renal tissues were collected from three randomly selected mice (*n* = 3) per group (Control, DKD, and SYFS) and subsequently submitted to Shanghai Majorbio Bio‐pharm Technology Co.,Ltd. for transcriptomic sequencing. The accession number for the RNA‐seq data reported in this paper is PRJNA1357101 (BioProject).

### 2.13. HK‐2 Cell Culture

The human proximal tubular epithelial cell line HK‐2 was kindly provided by Professor Weijing Liu from Dongzhimen Hospital, Beijing University of Chinese Medicine. The cells were cultured in Dulbecco’s Modified Eagle Medium (DMEM, 11966025, Gibco, California, USA) and F12 medium (11765054, Gibco, California, USA) supplemented with 10% fetal bovine serum (FBS, C04001‐500, Vivacell, Shanghai, China), a penicillin–streptomycin mixture (P1400, Solarbio, Beijing, China), and 5.5 mM glucose. HK‐2 cells were treated with culture medium containing various concentrations of SYFS lyophilized powder (0, 50, 100, 200, 400, 800, and 1600 μg/mL) for 24 h. Cell viability in each group was determined using a CCK‐8 assay kit (GK10001, GLPBIO, California, USA) to identify the optimal concentration and exposure duration of SYFS. Following the protocol described in reference [24], DKD model was established by exposing HK‐2 cells to 30 mmol/L glucose and 300 μmol/L palmitic acid (PA). The DKD model cells were treated with culture medium containing SYFS at concentrations of 50, 100, 150, 200, or 250 μg/mL for 24 h to determine the optimal treatment concentration. The cells were divided into three groups: (1) control group: HK‐2 cells; (2) DKD group: HK‐2 cells exposed to 30 mmol/L glucose and 300 μmol/L PA; and (3) SYFS group: HK‐2 cells treated with 30 mmol/L glucose, 300 μmol/L PA, and SYFS at the optimized concentration.

### 2.14. RNA Extraction and Real‐Time Quantitative Polymerase Chain Reaction (RT‐qPCR)

Gene expression was validated at both the animal and cellular levels using RT‐qPCR. Total RNA was extracted using an RNA extraction kit (R218‐50, JinbaiTe Biotechnology Co., Ltd., Beijing, China). mRNA was reverse‐transcribed into complementary DNA (cDNA) using the HiScript III All‐in‐one RT SuperMix Perfect for qPCR kit (R333‐01, Vazyme, Nanjing, China). Quantitative PCR amplification was performed using cDNA as the template and the Taq Pro Universal SYBR qPCR Master Mix (Q712, Vazyme, Nanjing, China). The amplification conditions were as follows: predenaturation at 95°C for 30 s, denaturation at 95°C for 10 s, annealing at 60°C for 30 s, for a total of 40 cycles. *β*‐Actin was used as the internal reference gene, and the relative expression levels of the target genes were calculated using the 2^−*ΔΔ*Ct^ method for statistical analysis. All primers were designed using the PrimerBank software and synthesized by Beijing Tsingke Biotech Co., Ltd. The primer sequences are listed in Supporting Information 2: Table S1.

### 2.15. Statistical Analysis

Statistical analysis was performed using R Studio 4.3.0. The normality of continuous data was assessed with the Shapiro–Wilk test. For data conforming to a normal distribution, comparisons between two groups were conducted using the Student’s *t*‐test, while comparisons across multiple groups were performed with one‐way ANOVA. Upon identifying a statistically significant overall difference (*p* < 0.05) in the ANOVA, post hoc pairwise comparisons were carried out using the LSD or Tukey’s method. For data that were not normally distributed, nonparametric tests were applied: the Wilcoxon rank‐sum test for comparing two groups, and the Kruskal–Wallis H test for comparing multiple groups, with subsequent Dunn’s test for pairwise comparisons when statistical significance was achieved.

## 3. Results

### 3.1. Components and Targets in SYFS Formula

Comprehensive scanning analysis in both positive and negative ion modes was conducted on the freeze‐dried powder samples of SYFS formula, and the total ion chromatogram (TIC) was obtained (Supporting Information 1: Figure S1). After manual verification and elimination of duplicate results, a total of 154 chemical components were identified in the SYFS formula, including flavonoids (naringenin), terpenoids (oleanonic acid), and alkaloids and their derivatives (palmatine), among others (Supporting Information 2: Table S2). The top 10 compounds by peak area were arginine, adenosine, mangiferin, tectorigenin‐7‐O‐*β*‐D‐glucoside, sorbitol, isomangiferin, tectorigenin, 5‐hydroxymethyl‐2‐furfural, guanine, and biochanin A. The regulatory targets of these 154 components were predicted using STITCH and SwissTarget databases. No regulatory targets were found for 17 components, while the remaining 137 components yielded a total of 4404 predicted targets. After removing duplicates, 994 regulatory targets of the components in SYFS formula were ultimately obtained (Supporting Information 2: Table S3).

### 3.2. Acquisition of DKD Targets

We merged the GSE96804 and GSE30528 datasets and employed batch effect removal to eliminate batch effects (Supporting Information 1: Figure S2). Differential expression analysis of the merged dataset identified 2,655 DEGs, comprising 1775 upregulated and 880 downregulated genes (Supporting Information 2: Table S4). The heatmap (Figure 2A) and volcano plot (Figure 2B) illustrate the clustering of DEGs and the differences between samples. Subsequently, a scale‐free coexpression network was constructed using WGCNA to identify the modules most closely associated with DKD. A “soft” *β* threshold of nine was selected based on scale independence and average connectivity (Figure 2C,D). A coexpression network was then constructed, with genes clustered to generate a clustering dendrogram for the DKD and control groups (Figure 2I), resulting in 19 gene coexpression modules of different colors, with a module merging threshold of 0.25 and a minimum size of 50, as shown in Figure 2E. Based on |Cor| ≥ 0.6, the lightcyan module (Figure 2F), brown module (Figure 2G), and lightgreen module (Figure 2H) were selected, yielding a total of 833 genes (Supporting Information 2: Table S5). The intersection of DEGs, key module genes from WGCNA, and target genes of components in SYFS yielded 39 genes (Figure 2J, Supporting Information 2: Table S6), which were predicted as potential targets of the SYFS formula for treating DKD.

Figure 2Identification of DKD targets: (A) volcano plot; (B) heatmap; (C–D) *β* = 9 is chosen as the soft threshold; (E) heatmap of the correlation between module genes and DKD, with correlation coefficients represented by color in the upper left triangle; *p*‐values are shown in the lower right corner; (F) lightcyan module; (G) brown module; (H) lightgreen module; (I) gene coexpression modules under the gene tree; (J) Venn diagram of intersecting genes.

**Figure:**
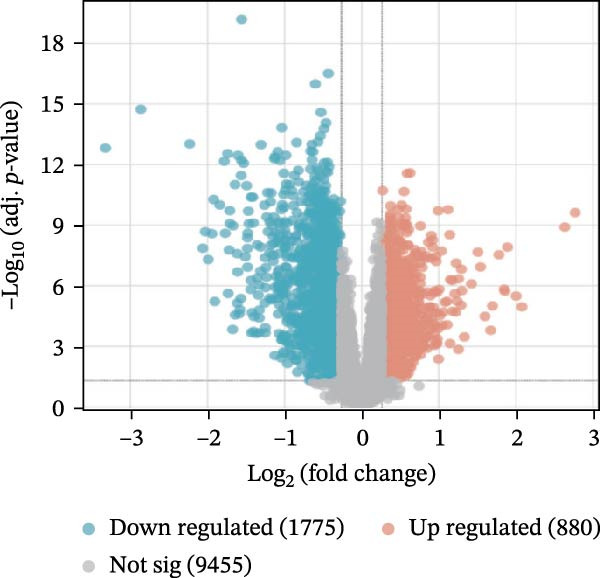
(A)

**Figure:**
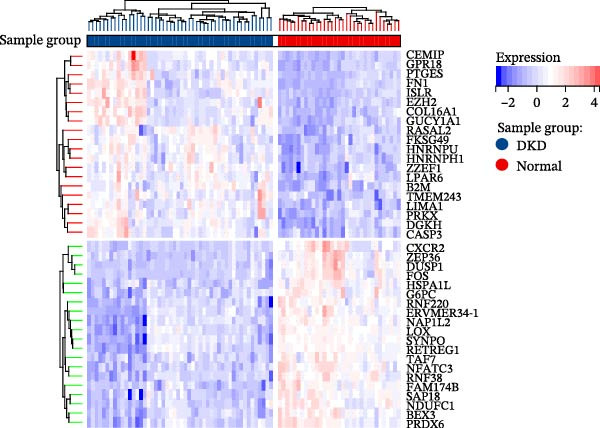
(B)

**Figure:**
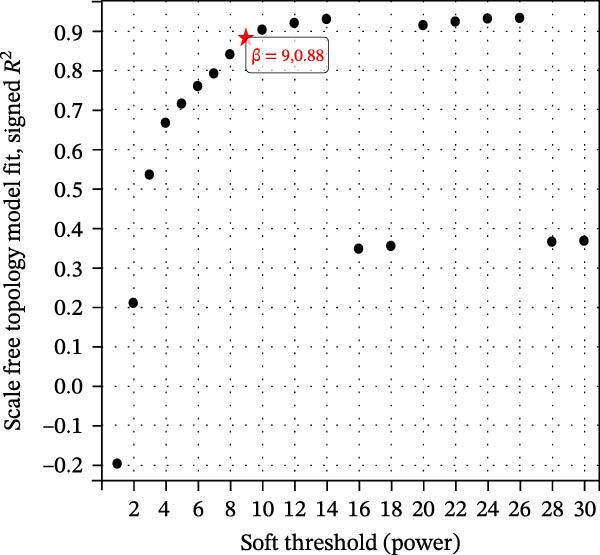
(C)

**Figure:**
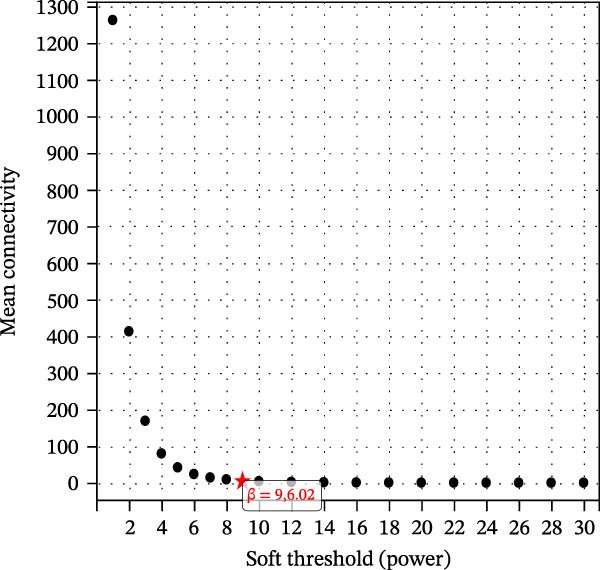
(D)

**Figure:**
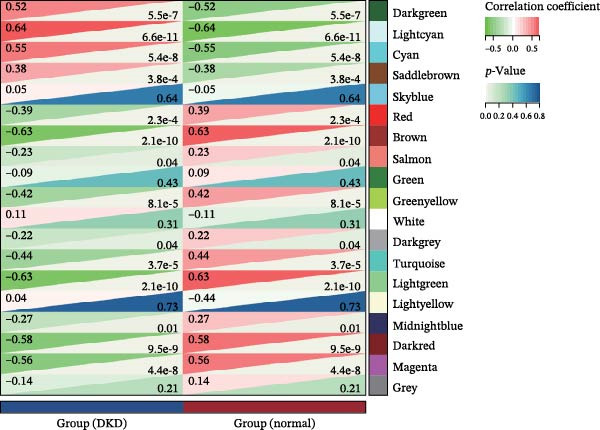
(E)

**Figure:**
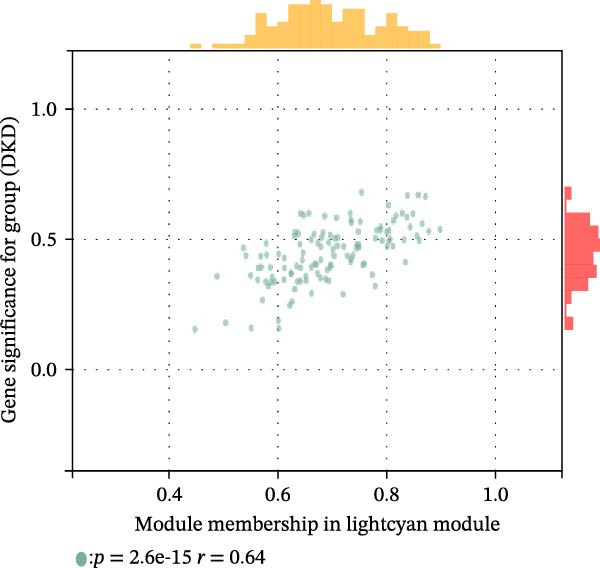
(F)

**Figure:**
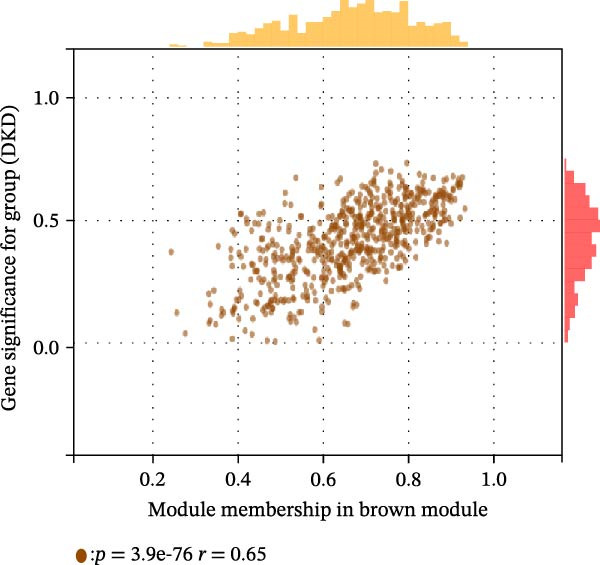
(G)

**Figure:**
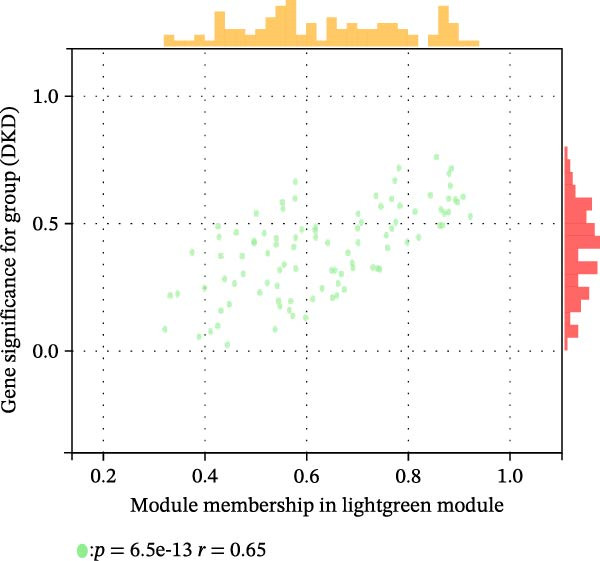
(H)

**Figure:**
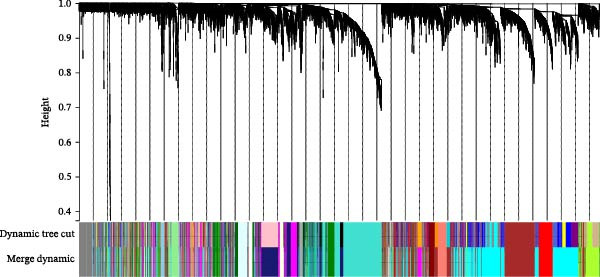
(I)

**Figure:**
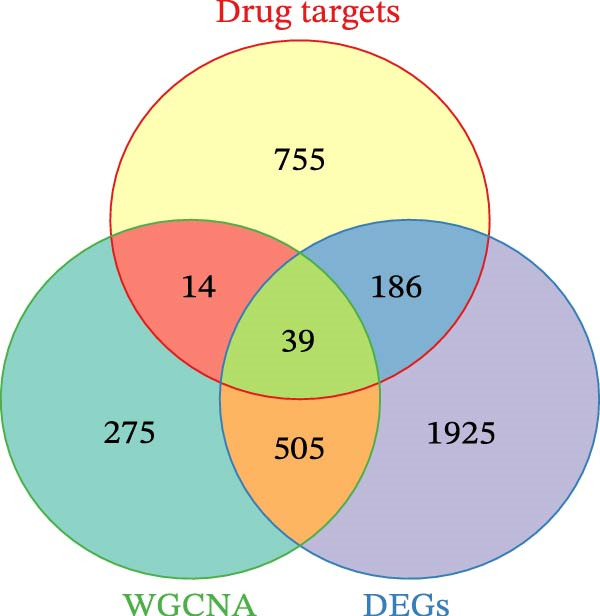
(J)

### 3.3. PPI Network and Component‐Target Network

Next, 39 potential targets were analyzed, and Figure 3A shows the chromosomal regions of these genes, which facilitate the identification and analysis of similarities and differences in comparative genomics studies. Subsequently, PPI network is shown in Figure 3B. The higher the Degree of the target protein, the larger and darker its node, indicating that the protein plays a more critical role through interactions with other targets. Table 3 displays the top 20 proteins identified as having the highest degree values. Furthermore, we constructed a component‐target network (Figure 3C). Components with the highest degree values, such as naringenin chalcone, palmatine, oleanonic acid, *β*‐elemonic acid, and naringenin, are the main component for the treatment of DKD with SYFS formula (Table 4).

Figure 3PPI network and component‐target network: (A) chromosomal location map of 39 genes; (B) PPI network; (C) component‐target network. Green circles represent components of SYFS formula, pink diamonds represent potential targets, blue triangles represent DKD, and connecting lines indicate associations between nodes.

**Figure:**
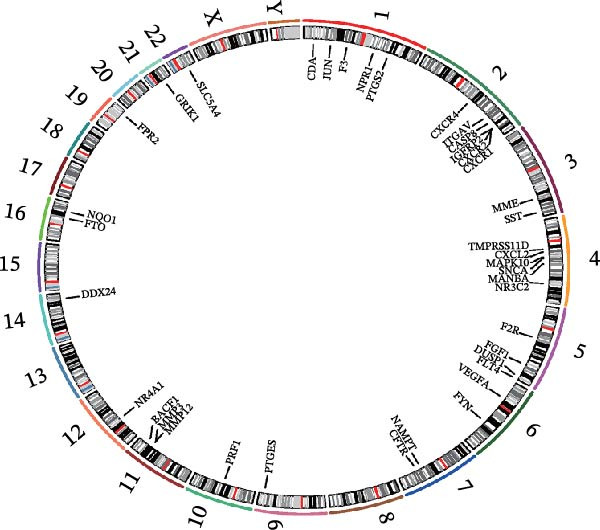
(A)

**Figure:**
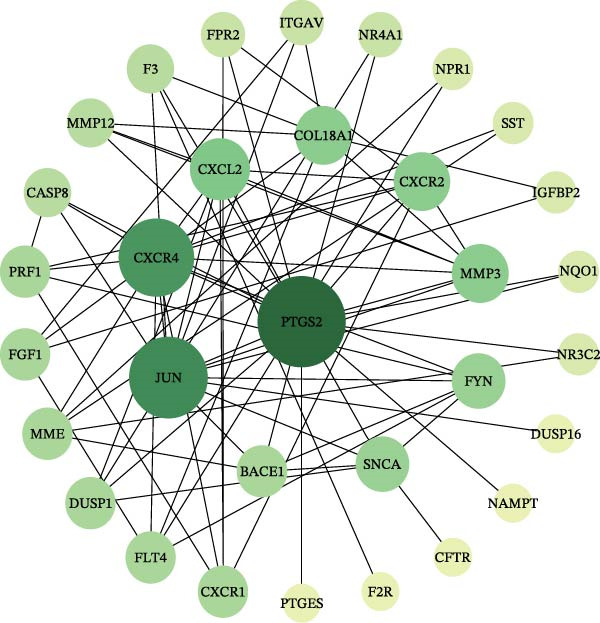
(B)

**Figure:**
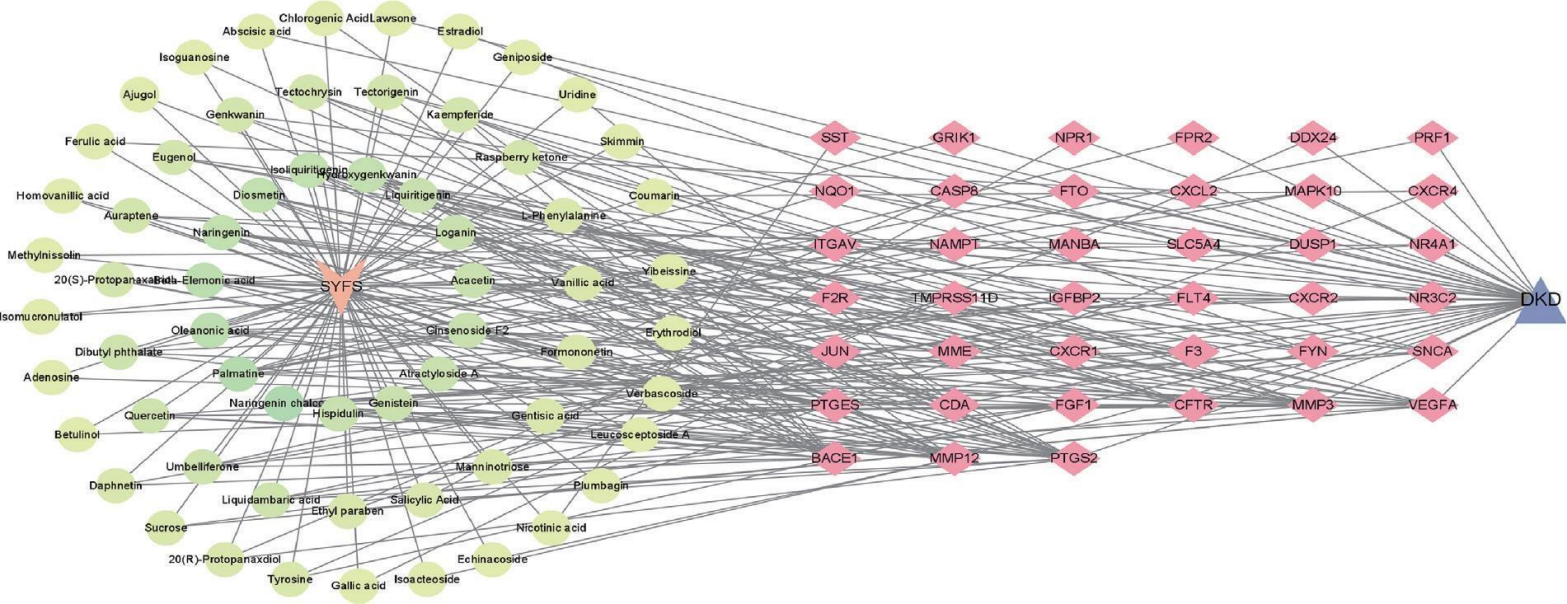
(C)

**Table 3:** Information about the top 20 targets.

Target name	Target acronym	Degree	Betweenness	Closeness
Prostaglandin‐endoperoxide synthase 2	PTGS2	21	0.046	0.452
Matrix metallopeptidase 12	MMP12	19	0.040	0.444
*β*‐Secretase 1	BACE1	17	0.032	0.436
Vascular endothelial growth factor A	VEGFA	13	0.021	0.422
Matrix metallopeptidase 3	MMP3	13	0.018	0.422
Cystic fibrosis transmembrane conductance regulator	CFTR	11	0.011	0.415
Cytidine deaminase	CDA	7	0.013	0.402
Prostaglandin E synthase	PTGES	7	0.010	0.402
Fibroblast growth factor 1	FGF1	7	0.009	0.399
α‐Synuclein	SNCA	7	0.006	0.402
Fyn proto‐oncogene tyrosine kinase	FYN	6	0.010	0.399
C‐X‐C motif chemokine receptor 1	CXCR1	6	0.004	0.399
Tissue factor	F3	6	0.004	0.399
Membrane metallo‐endopeptidase	MME	5	0.003	0.396
C‐X‐C motif chemokine receptor 2	CXCR2	4	0.004	0.393
Nuclear receptor subfamily 3 group C member 2	NR3C2	4	0.004	0.393
Fms‐like tyrosine kinase 4	FLT4	4	0.004	0.393
Insulin‐like growth factor binding protein 2	IGFBP2	4	0.003	0.393
Transmembrane serine protease 11D	TMPRSS11D	4	0.004	0.393
c‐Jun proto‐oncogene	JUN	4	0.004	0.393

**Table 4:** Information on the top 20 components.

Rank	Ingredient name	Degree	Betweenness centrality	Closeness centrality
1	Naringenin chalcone	10	0.014	0.456
2	Palmatine	9	0.027	0.452
3	Oleanonic acid	8	0.017	0.448
4	*β*‐Elemonic acid	8	0.014	0.448
5	Naringenin	7	0.007	0.444
6	Diosmetin	7	0.006	0.444
7	Isoliquiritigenin	7	0.007	0.444
8	Hydroxygenkwanin	7	0.006	0.444
9	Genistein	6	0.006	0.440
10	Hispidulin	6	0.004	0.440
11	Liquiritigenin	6	0.005	0.440
12	Acacetin	6	0.006	0.440
13	Ginsenoside F2	6	0.006	0.440
14	Atractyloside A	6	0.010	0.440
15	Loganin	6	0.010	0.440
16	Liquidambaric acid	5	0.005	0.436
17	Dibutyl phthalate	5	0.008	0.436
18	Quercetin	5	0.003	0.436
19	Umbelliferone	5	0.007	0.436
20	Eugenol	4	0.006	0.433

### 3.4. Enrichment Analysis and Component‐Target‐Pathway Network

The GO enrichment analysis revealed that the biological processes (BP) were enriched in: response to amyloid‐*β*, cell chemotaxis, and neuroinflammatory response. The cellular component (CC) included secretory granule membrane, membrane raft, and early endosome. The molecular functions (MF) included G protein‐coupled peptide receptor activity, chemokine binding, and immune receptor activity (Figure 4A). Furthermore, KEGG pathway enrichment analysis were primarily enriched in complement and coagulation cascade, Apoptosis, MAPK, VEGF, and TNF signaling pathway (Figure 4B). Based on the top 10 pathways identified through KEGG analysis, we developed a component‐target‐pathway network diagram for DKD treatment with the SYFS formula (Figure 4C).

Figure 4Enrichment analysis and component‐target‐pathway network: (A) the bar plot of GO analysis; (B) the bubble plot of KEGG analysis; (C) component‐target‐pathway network. The blue rhombus represents the components of SYFS formula, orange circles denote potential targets, green inverted triangles indicate signaling pathways, and red hexagons represent DKD disease.

**Figure:**
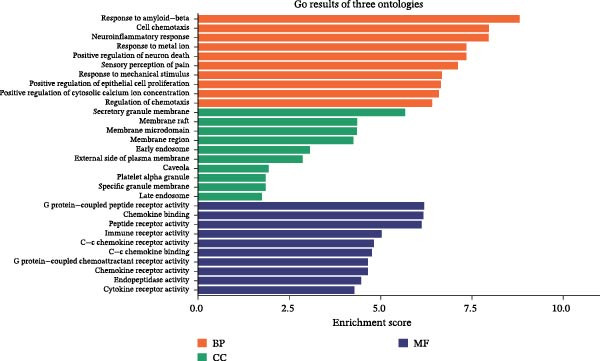
(A)

**Figure:**
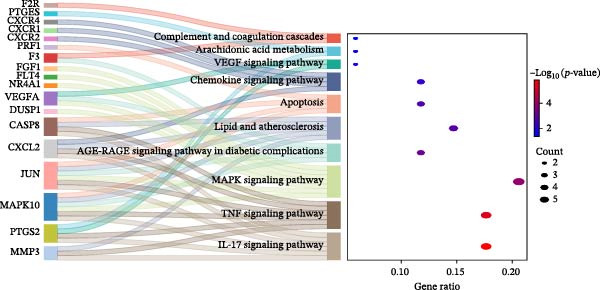
(B)

**Figure:**
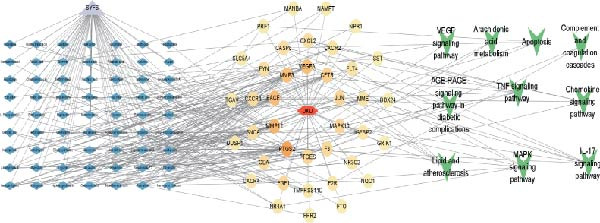
(C)

### 3.5. ML for Hub Genes Screening

To further screen potential targets, 8 ML prediction models were constructed, including RF, SVM, GLM, GBM, KNN, NNET, LASSO, and DT. A comprehensive comparative analysis was conducted on the ROC curves, residual box plots, and residual reverse cumulative distribution curves of each model (Figure 5A–C). The results showed that the SVM, NNET, and LASSO models exhibited superior classification performance in this study’s dataset, with higher AUC values, lower residual distributions, and better error accumulation characteristics. This suggests that these models have high stability and accuracy in distinguishing between different groups. Based on the above results, we further combined the importance scores of feature genes in the SVM, NNET, and LASSO models, selecting the top 10 candidate genes from each model. The intersection of the results from the three models was taken to reduce the bias of any single model. Ultimately, 5 key genes were identified, including MMP3, MMP12, PTGES, SST, and DUSP1 (Figure 5D,E). These hub genes are closely related to each other (Figure 5F–G). MMP3, MMP12, and PTGES are increased in DKD group, while SST and DUSP1 are decreased (*p* < 0.05) (Figure 5H).

Figure 5ML screening of hub genes: (A) ROC curve; (B) residual box plot; (C) residual reverse cumulative distribution plot; (D) important feature genes; (E) Venn diagram of three machine learning methods; (F–G) hub gene correlations; (H) expression of hub genes.

**Figure:**
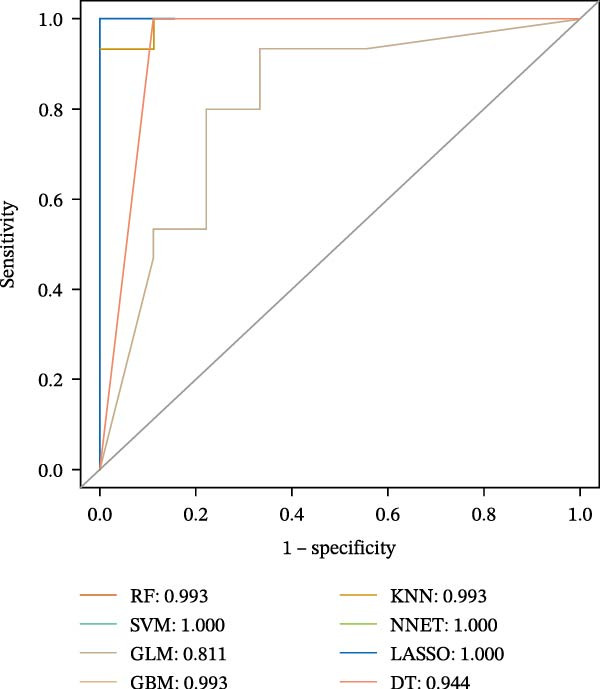
(A)

**Figure:**
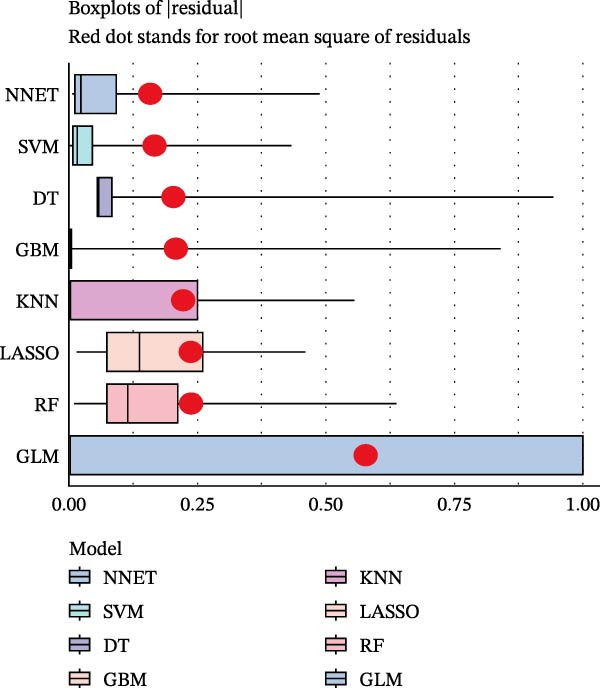
(B)

**Figure:**
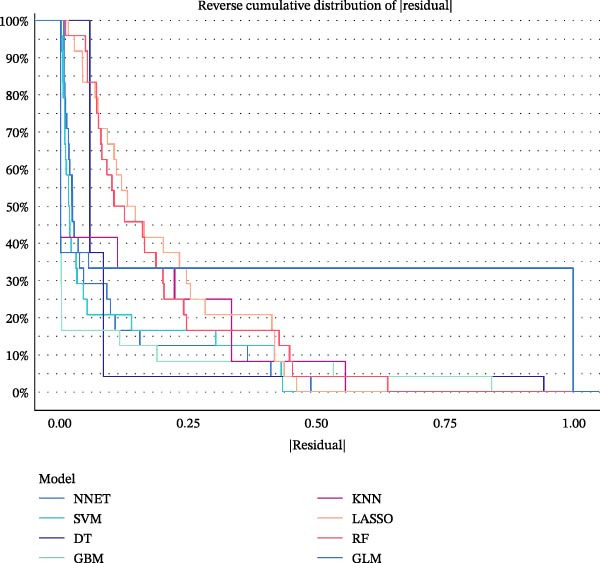
(C)

**Figure:**
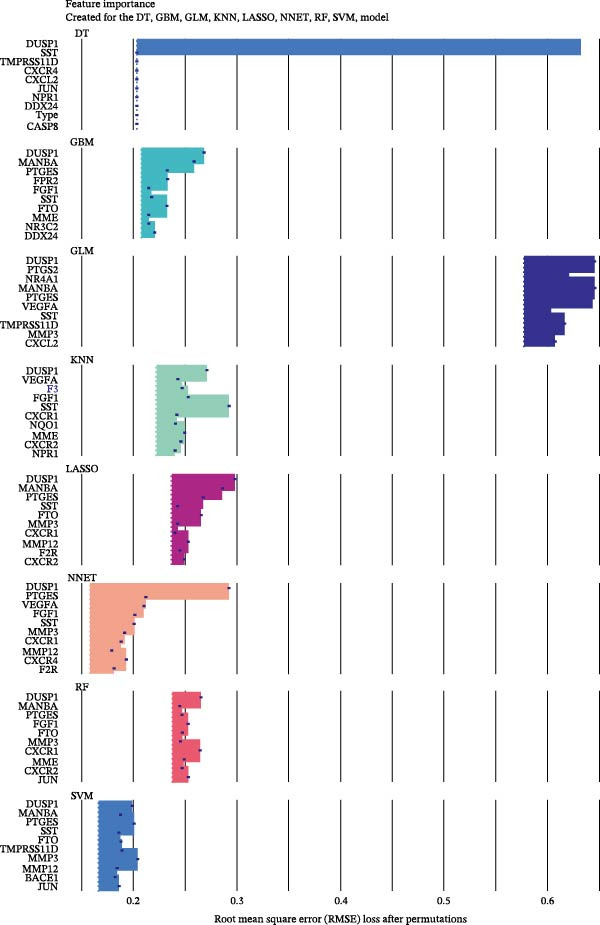
(D)

**Figure:**
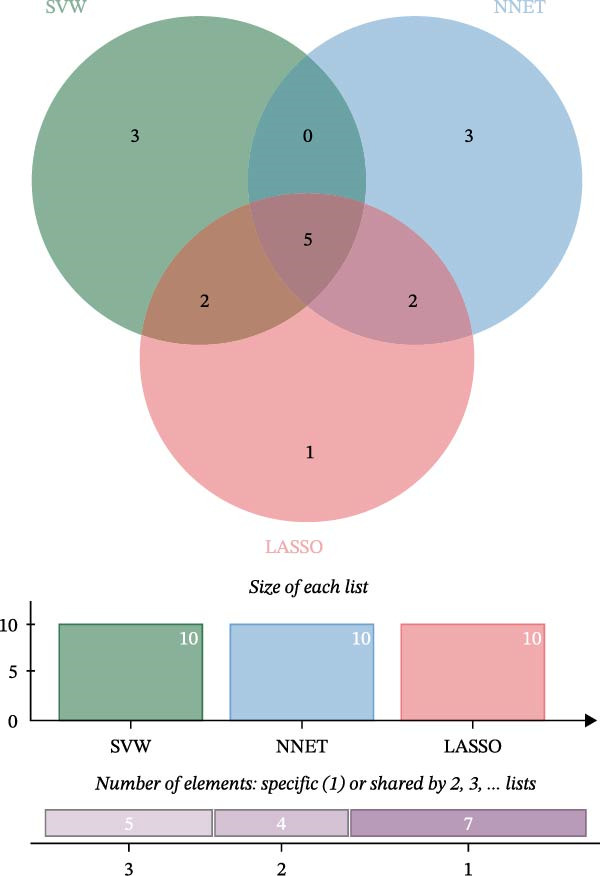
(E)

**Figure:**
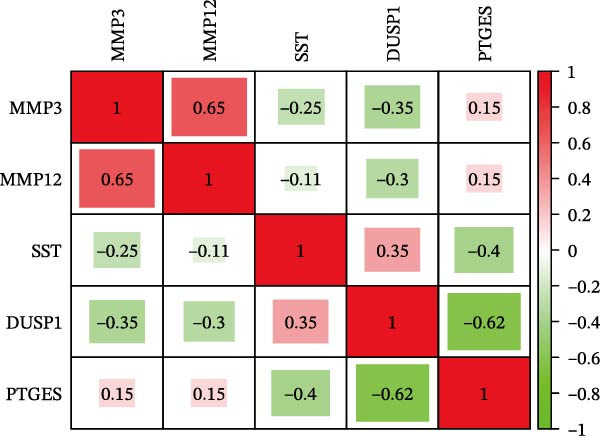
(F)

**Figure:**
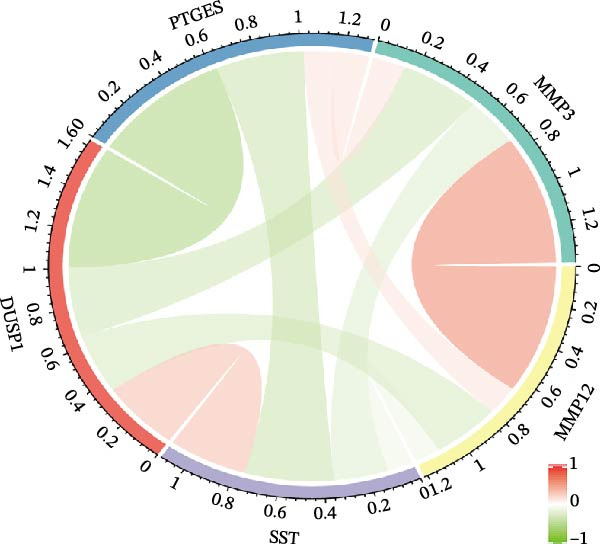
(G)

**Figure:**
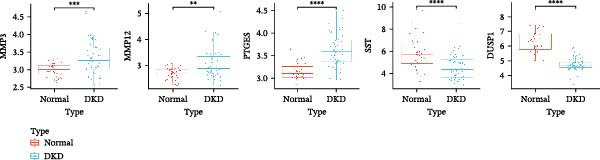
(H)

### 3.6. Construction of Nomogram

We constructed a Nomogram model using selected hub genes (Figure 6A). Figure 6B shows that the C‐index is 0.983, indicating high accuracy in the model’s predictive capability. The AUC of hub genes ranges from 0.708 to 0.983, demonstrating good discriminatory ability between DKD and normal group (Figure 6C). In training set, the risk score of DKD group was markedly higher than normal group (*p* < 0.001), with an AUC of 0.963 (Figure 6C,D). The same trend was observed in the GSE30529 dataset (Figure 6E,F) and Woroniecka Diabetes Glom dataset (Figure 6G,H), with AUC of 0.883 and 0.966, respectively. In the Woroniecka Diabetes Glom dataset, further analysis of correlation between hub genes and renal function showed that risk score was negatively correlated with GFR, with GFR decreasing as the risk score increased (*r* = −0.67, *p*  < 0.01) (Figure 6I). Among the hub genes, MMP3, MMP12, and PTGES showed no correlation with GFR (Figure 6J–L). In contrast, SST (*r* = 0.54, *p*  < 0.01) and DUSP1 (*r* = 0.5, *p* = 0.021) exhibited positive correlations with GFR (Figure 6M,N).

Figure 6Construction of nomogram: (A) nomogram; (B) calibration curve; (C) ROC curve of training set; (D) risk scores in training set; (E) ROC curve in GSE30529 dataset; (F) risk scores in GSE30529 dataset; (G) ROC curve in Woroniecka Diabetes Glom dataset; (H) risk scores in Woroniecka Diabetes Glom dataset; (I) correlation scatter plot of risk scores and GFR in Woroniecka Diabetes Glom dataset; (J‐N) correlation scatter plots of hub genes and GFR in Woroniecka Diabetes Glom dataset.

**Figure:**
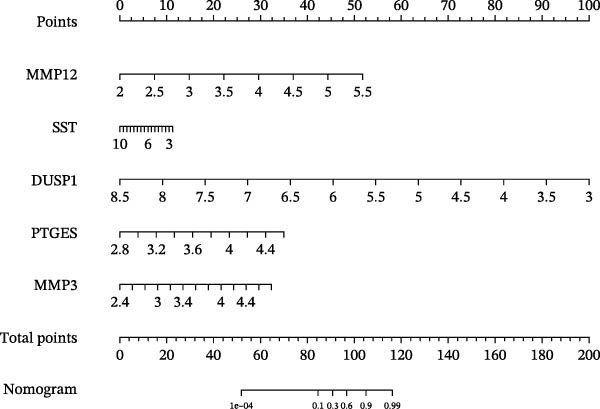
(A)

**Figure:**
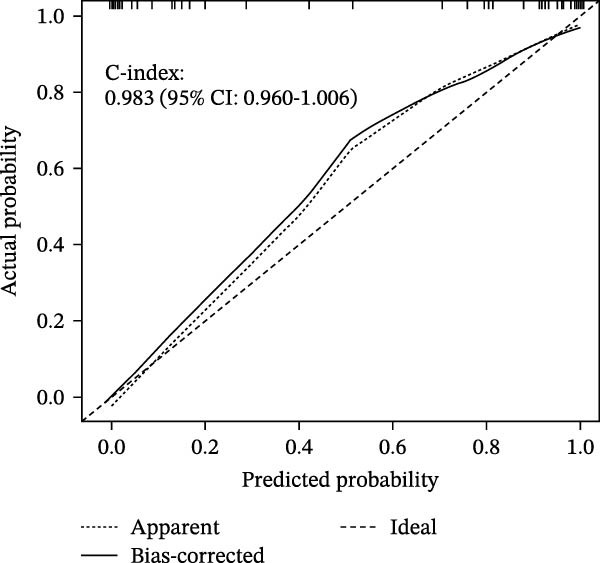
(B)

**Figure:**
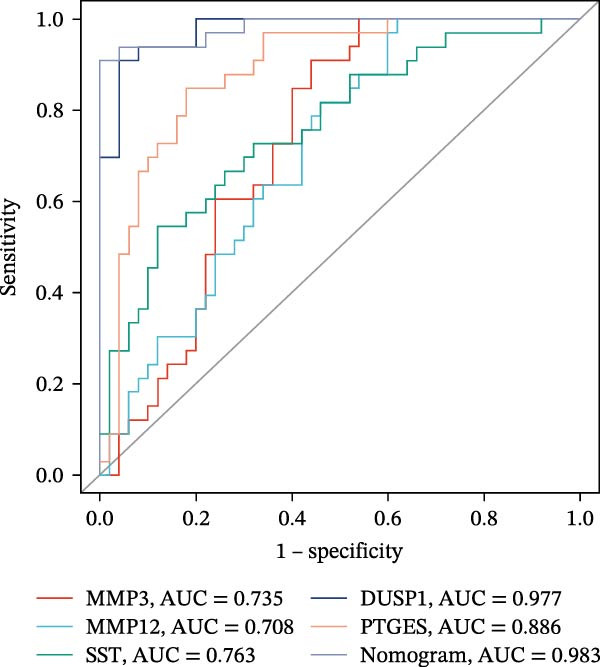
(C)

**Figure:**
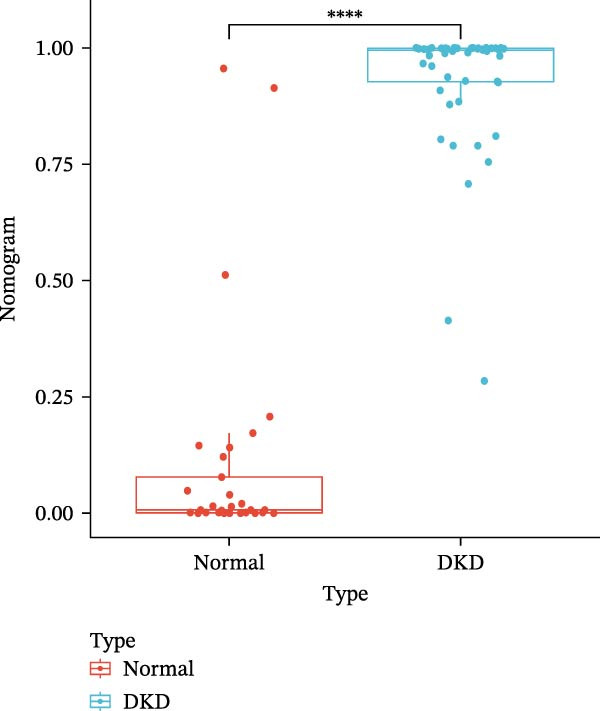
(D)

**Figure:**
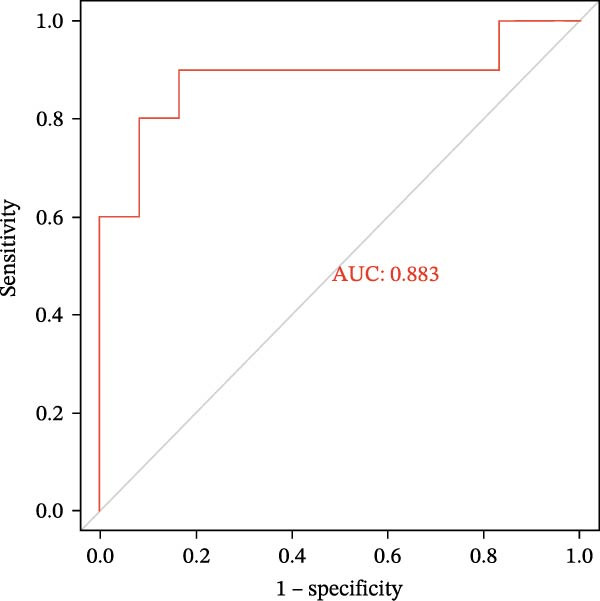
(E)

**Figure:**
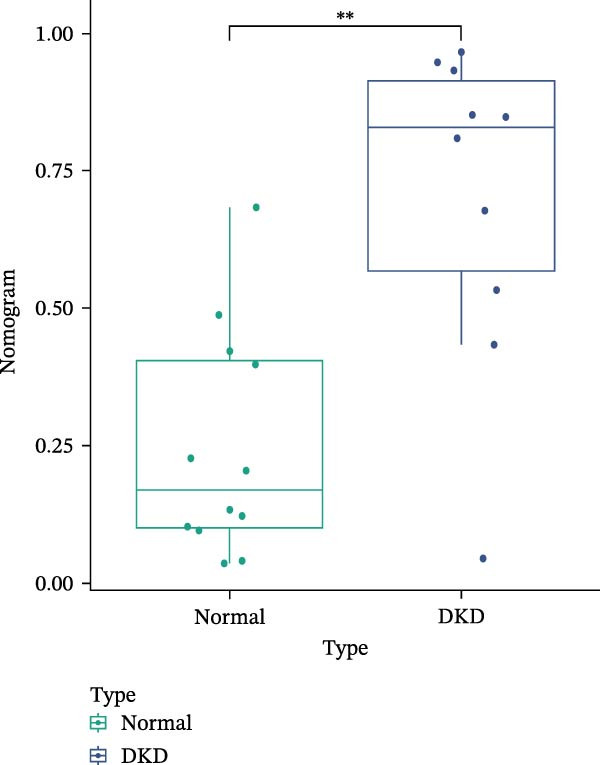
(F)

**Figure:**
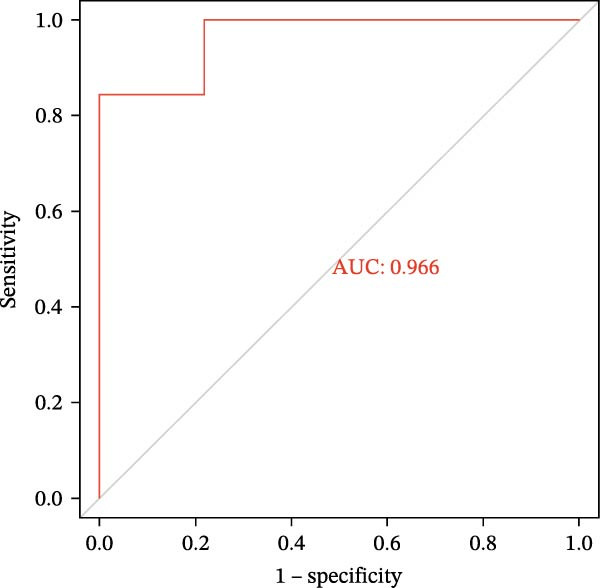
(G)

**Figure:**
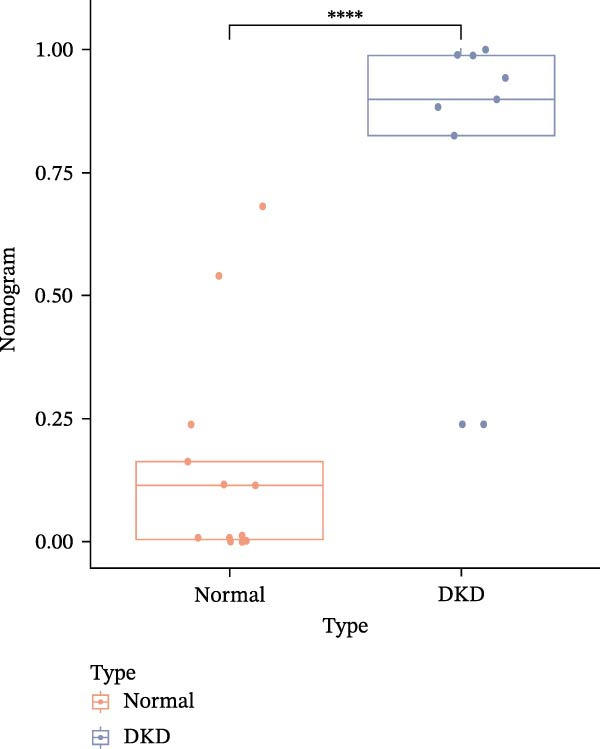
(H)

**Figure:**
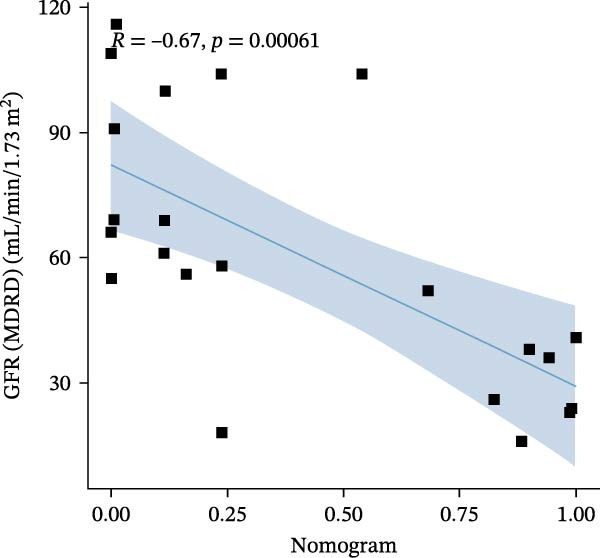
(I)

**Figure:**
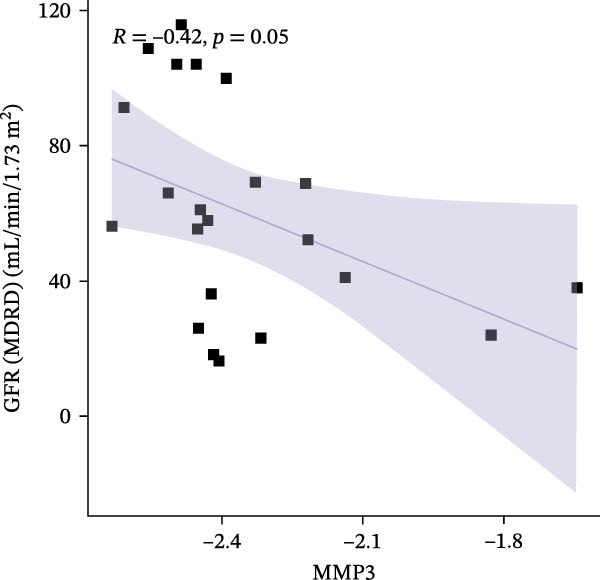
(J)

**Figure:**
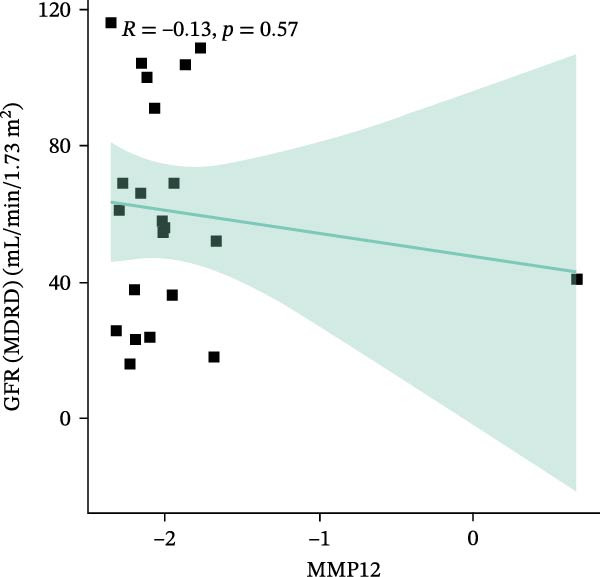
(K)

**Figure:**
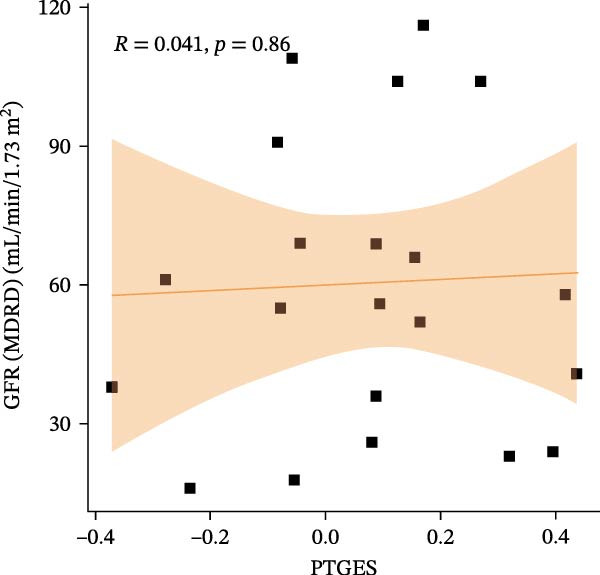
(L)

**Figure:**
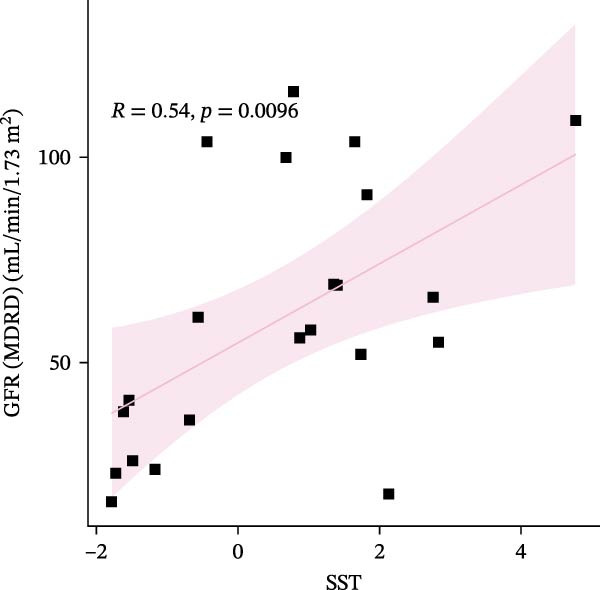
(M)

**Figure:**
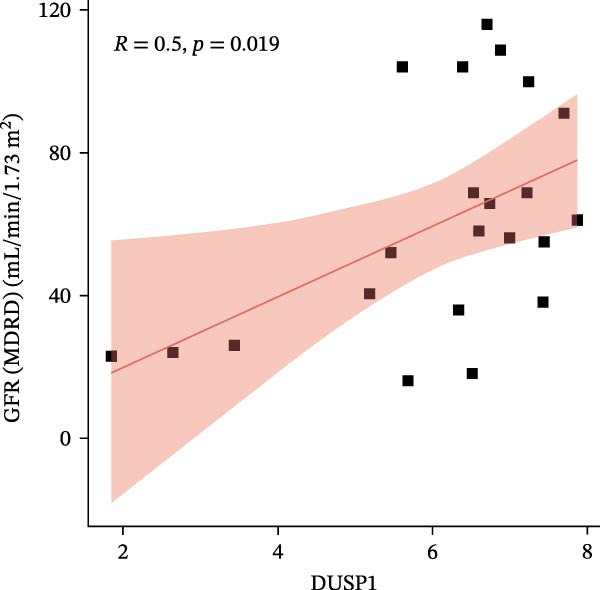
(N)

### 3.7. Immune Infiltration Analysis

In order to explore immune microenvironment of DKD, CIBERSORT algorithm and ssGSEA were used to analyze the level of immune cell levels (Figure 7A). The CIBERSORT analysis revealed a significant increase in multiple immune cell subsets in the DKD group, notably memory B cells, *γδ* T cells, and M1 and M2 macrophages, suggesting their potential involvement in the pathogenesis of DKD. Further correlation analysis demonstrated associations between specific immune cell types and genes: MMP3 was correlated with Tregs (regulatory T cells) and resting dendritic cells; MMP12 with Tregs and activated NK cells; PTGES with memory B cells and M2 macrophages; SST with neutrophils; and DUSP1 with neutrophils, activated mast cells, and M2 macrophages (Figure 7B). Similar results were further validated by ssGSEA (Figure 7C,D).

Figure 7Immune infiltration analysis. (A–B) CIBERSORT: (A) box plots of immune cells; (B) relation between hub genes and immune cells. (C–D) ssGSEA: (C) box plots of immune cells; (D) relation between hub genes and immune cells.

**Figure:**
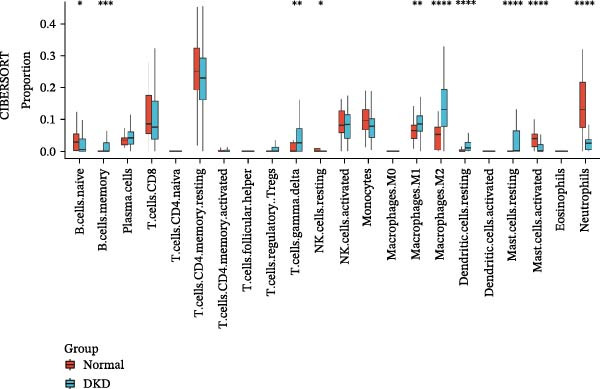
(A)

**Figure:**
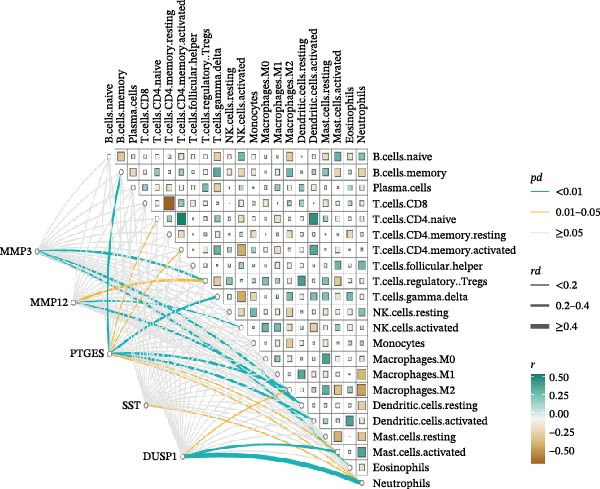
(B)

**Figure:**
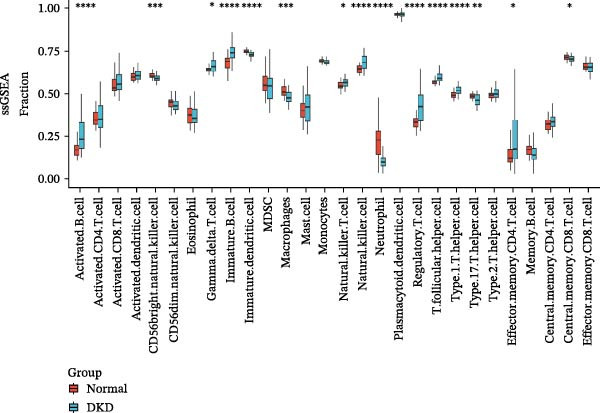
(C)

**Figure:**
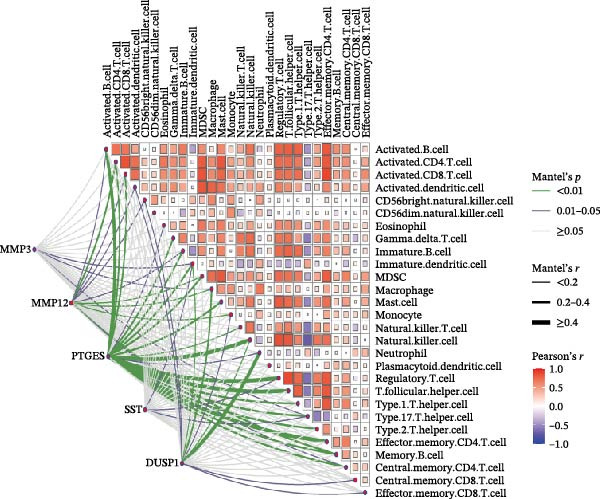
(D)

### 3.8. Consensus Clustering

The results of consensus clustering analysis indicated that the optimal number of clusters was *k* = 2, as shown by the consensus matrix, consensus CDF plot, and delta area plot (Figure 8A–C). Accordingly, 50 DKD patients were classified into two clusters, C1 (29 patients) and C2 (21 patients). Further PCA analysis showed a significant separation between C1 and C2 (Figure 8D). Immune infiltration analysis revealed a significant increase in immune cells, including naive B cells, plasma cells, Tregs, activated NK cells, Macrophages M1, and resting endritic cells, in the C2 (*p* < 0.05), indicating that C2 had a higher state of immune infiltration (Figure 8E). Box plots of hub genes also clearly demonstrated significant differences between two clusters (Figure 8F). Specifically, MMP3 and MMP12 were significantly lower and PTGES was significantly higher in C1. GSVA analysis was employed to evaluate biological function differences across the two clusters. Pathways such as complement and coagulation cascades, and ECM receptor interaction were upregulated in C1, while phosphate metabolism, regulation of autophagy, and insulin signaling pathway were upregulated in C2 (Figure 8G).

Figure 8Consensus clustering analysis: (A) consensus clustering matrix (*k* = 2); (B) CDF curve; (C) area under the CDF curve; (D) PCA plot; (E) immune infiltration analysis of the two clusters; (F) expression box plot of hub genes in the two clusters; (G) GSVA analysis of the two clusters.

**Figure:**
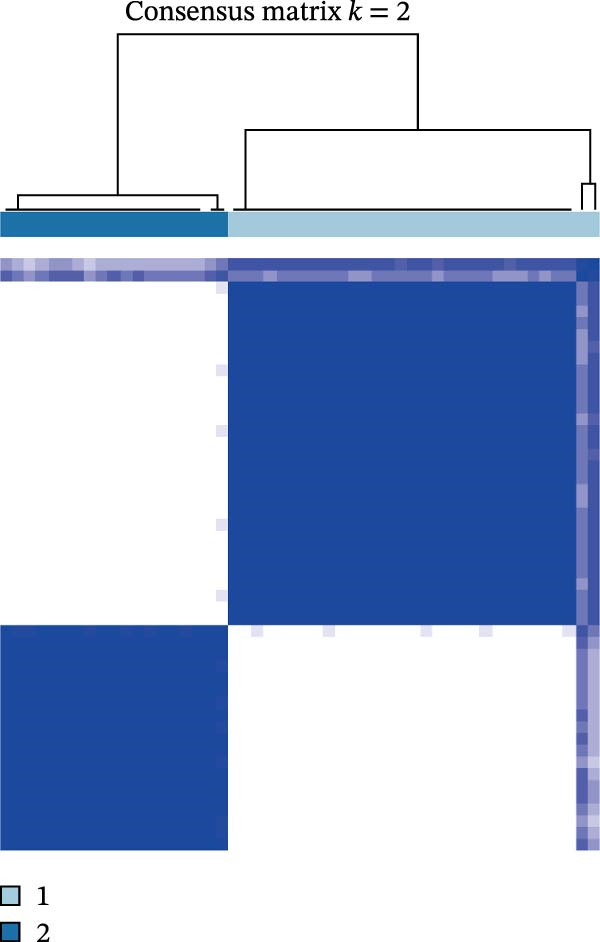
(A)

**Figure:**
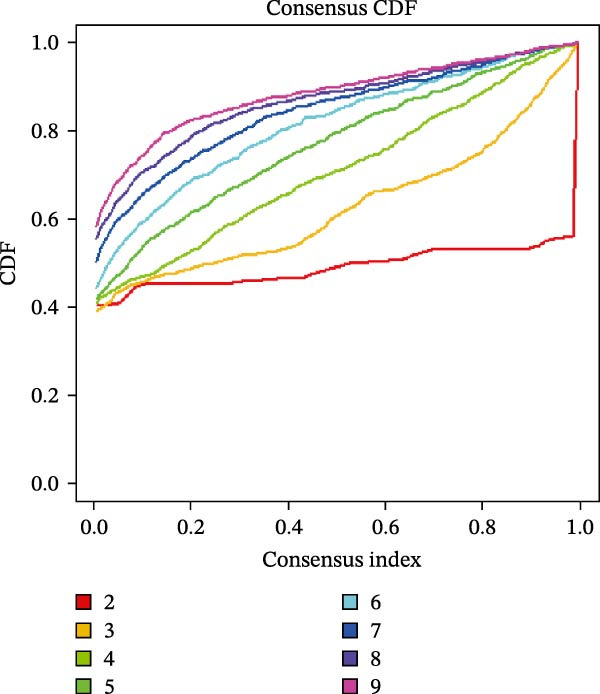
(B)

**Figure:**
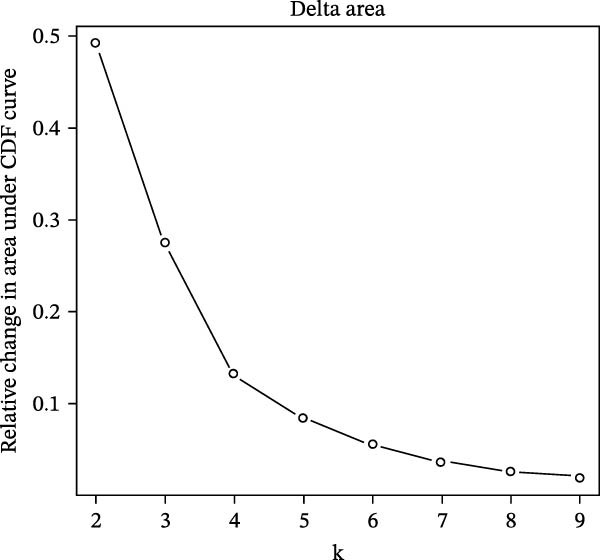
(C)

**Figure:**
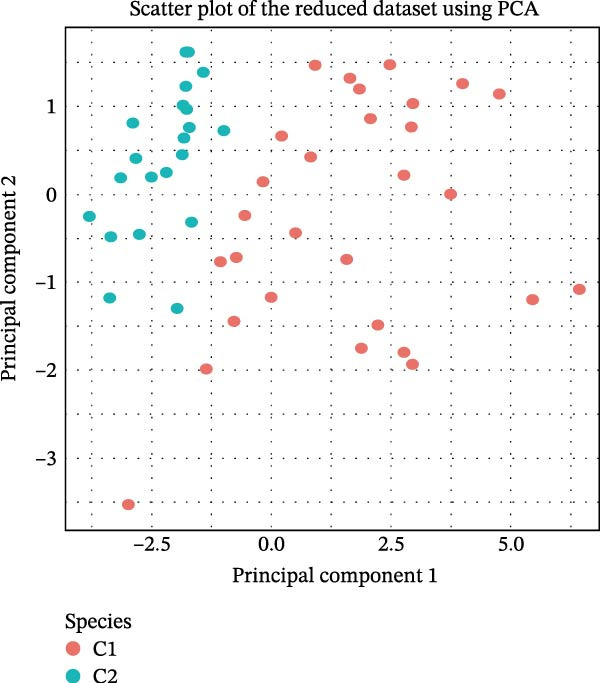
(D)

**Figure:**
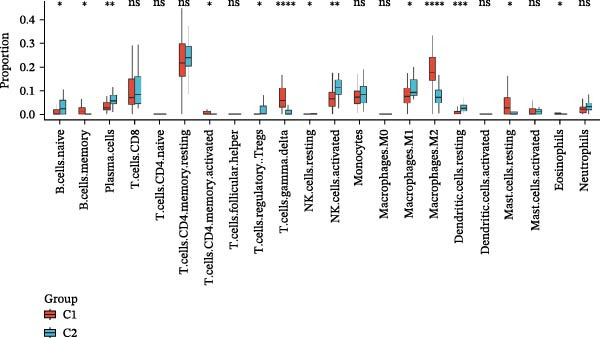
(E)

**Figure:**
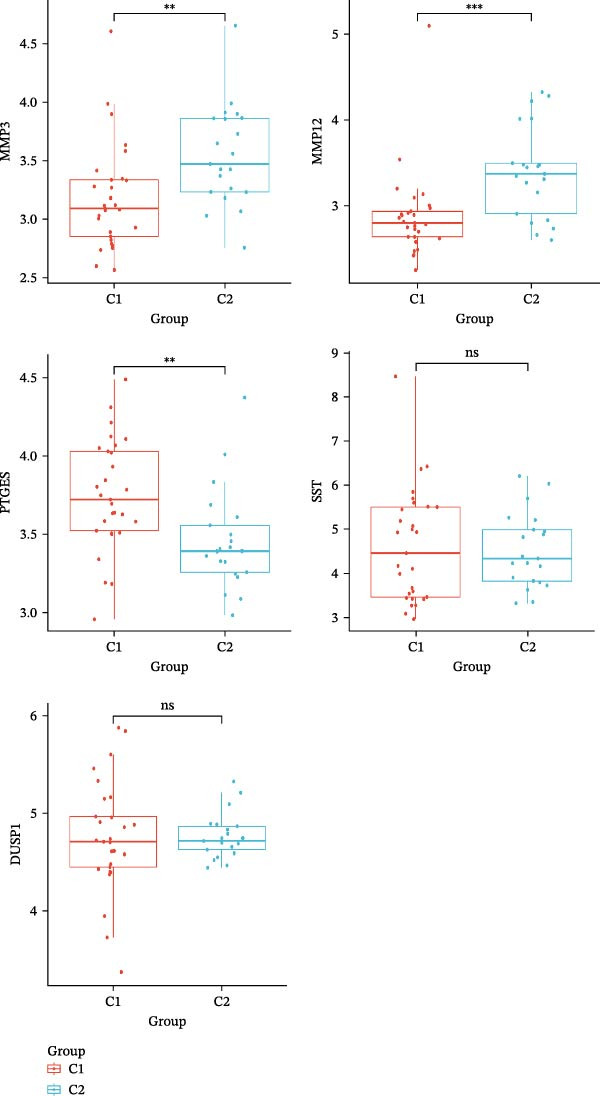
(F)

**Figure:**
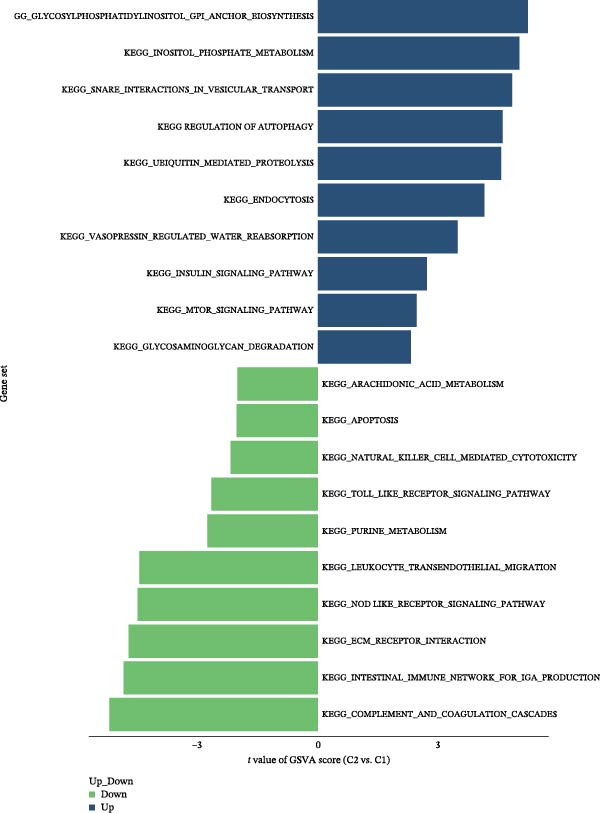
(G)

### 3.9. Molecular Docking and MD Simulation

To further validate the predictive power of the network, molecular docking was performed between five hub genes and top five ingredients: naringenin chalcone, palmatine, oleanonic acid, *β*‐elemonic acid, and naringenin. Figure 9A displays the binding energy heatmap, listing the binding energies of five core components with various targets. The lower the binding energy value, the more stable the binding. The binding energy between MMP3 and naringenin is −9.5 kcal/mol (Figure 9B), between MMP12 and naringenin is −9.3 kcal/mol (Figure 9C), and between DUSP1 and oleanonic acid is −9.1 kcal/mol (Figure 9D), also showing strong binding capability.

Figure 9Molecular docking results: (A) binding energy heatmap of core components of SYFS formula with target proteins; (B) MMP3‐naringenin; (C) MMP12‐naringenin; (D) DUSP1‐oleanonic acid.

**Figure:**
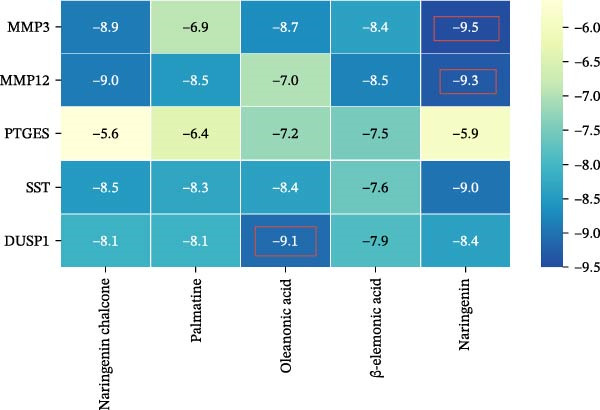
(A)

**Figure:**
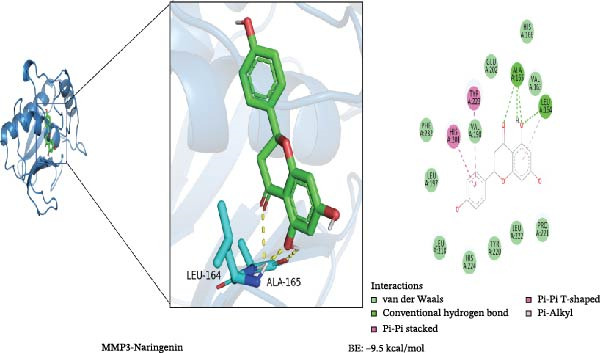
(B)

**Figure:**
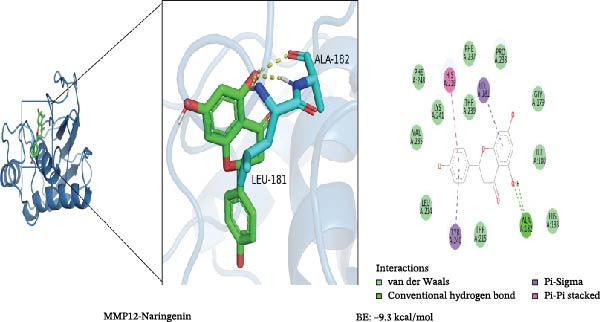
(C)

**Figure:**
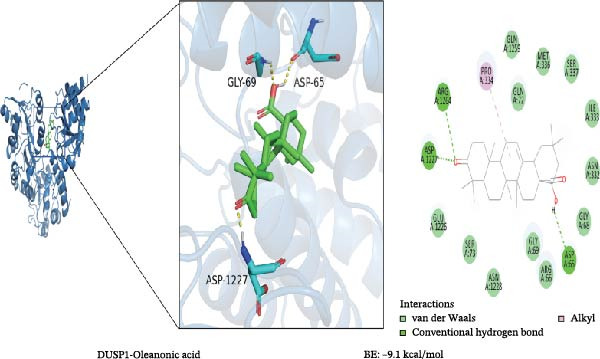
(D)

The binding energy between MMP3 and naringenin was the lowest among all tested complexes, and its stability was further verified through molecular dynamics simulations. The root mean square deviation (RMSD) analysis (Figure 10A) showed that the system reached equilibrium after ~60 ns, maintaining stability within 3–3.5 Å, indicating that the complex adopted a stable conformation. The radius of gyration (Rg) (Figure 10B) exhibited only minor fluctuations, suggesting that the protein maintained a compact structural organization. The solvent‐accessible surface area (SASA) (Figure 10C) remained nearly constant throughout the simulation, implying that ligand binding caused negligible conformational alteration to the protein. The number of hydrogen bonds (Figure 10D) fluctuated between 0 and 3, averaging around 2, which indicated persistent and stable hydrogen bond interactions. The residue root mean square fluctuation (RMSF) (Figure 10E) values were mostly below 2.3 Å, reflecting low overall flexibility and high structural stability. The MM‐PBSA binding free energy results (Figure 10F) revealed that van der Waals (*Δ*EvdW) and electrostatic (*Δ*Eele) interactions were the primary driving forces, with both polar (*Δ*GPB) and nonpolar (*Δ*GSA) solvation energies contributing moderately to the overall binding affinity. The per‐residue free energy decomposition (Figure 10G) identified several critical residues that reinforced complex stability through hydrogen bonding and hydrophobic contacts. The free energy landscape based on Rg and RMSD parameters displayed a distinct global energy minimum corresponding to the thermodynamically most stable conformational state. Furthermore, the electrostatic potential surface (Figure 10H,I) indicated that negative potentials were concentrated near electronegative atoms (electrophilic sites), revealing a clear molecular polarity and well‐defined interaction interface.

Figure 10Molecular dynamics simulation (MMP3 and naringenin): (A) RMSD; (B) Rg; (C) SASA; (D) number of hydrogen bonds; (E) RMSF; (F) binding free energy (MM‐PBSA analysis); (G) per‐residue energy decomposition; (H) free energy landscape (2D); (I) free energy landscape (3D).

**Figure:**
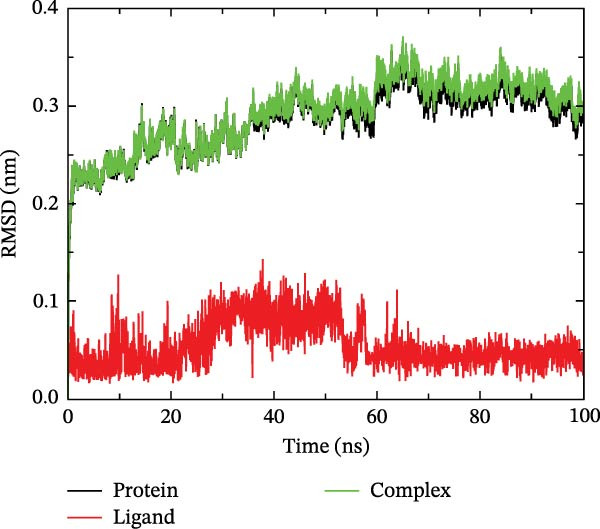
(A)

**Figure:**
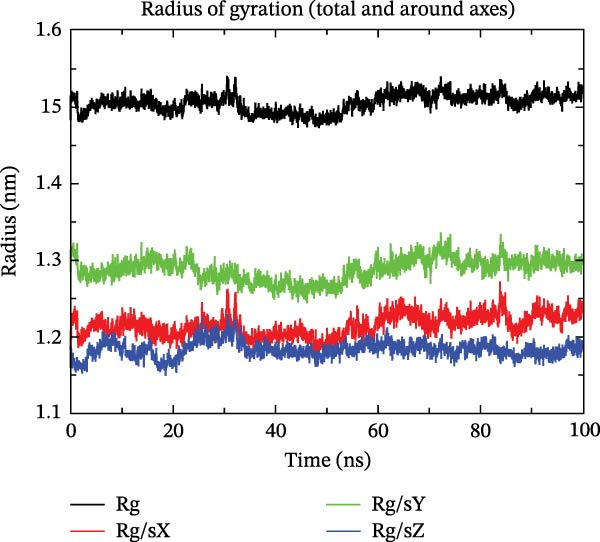
(B)

**Figure:**
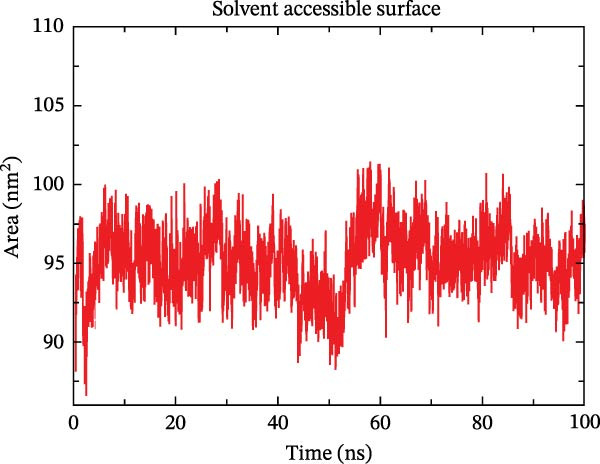
(C)

**Figure:**
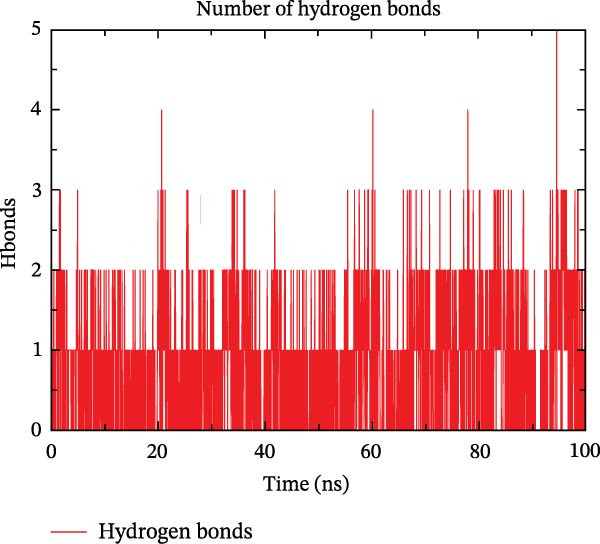
(D)

**Figure:**
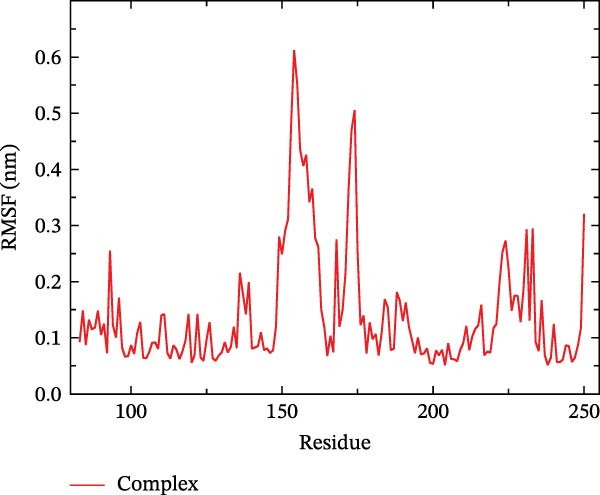
(E)

**Figure:**
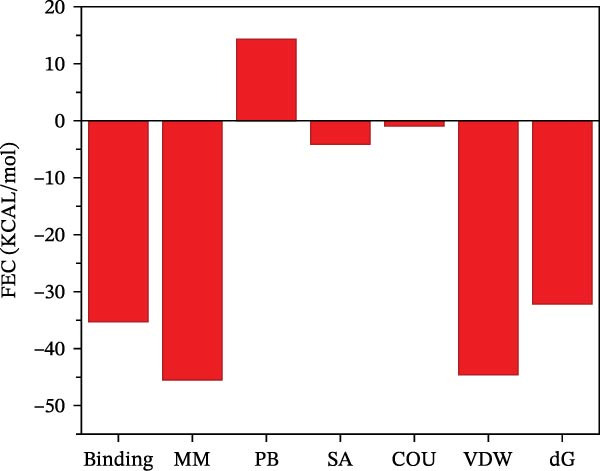
(F)

**Figure:**
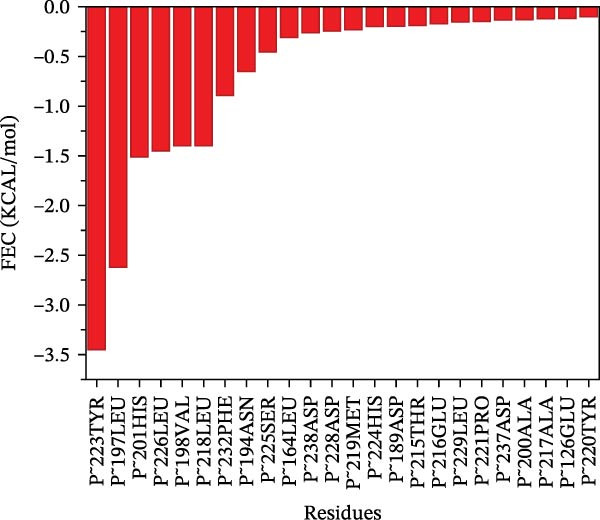
(G)

**Figure:**
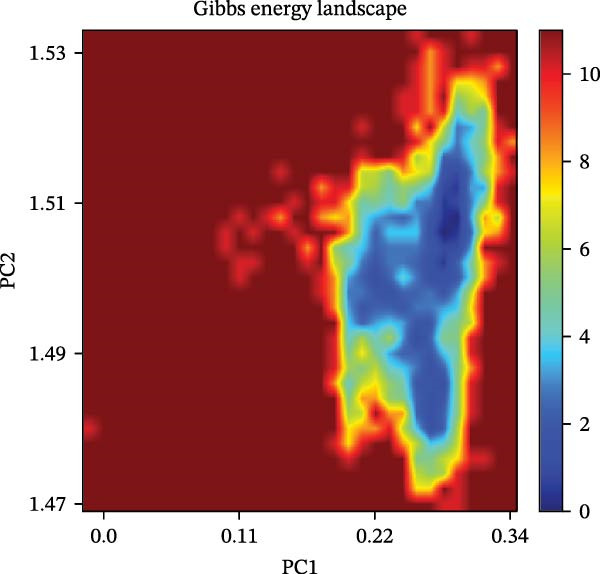
(H)

**Figure:**
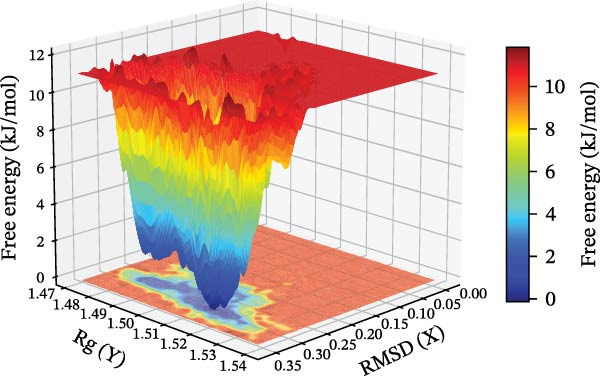
(I)

### 3.10. Transcriptomic Validation

Figure 11A systematically compared the pathological features of renal tissues in Control, DKD, SYFS, and DA groups through HE, PAS, and Masson staining. Pathological staining showed normal glomerular and tubular structures in the Control group, while the DKD group exhibited disordered renal tissue structure, glomerular hypertrophy, capillary loop proliferation, expanded renal capsule cavity, and irregular dilation of renal tubular lumens. Compared with DKD group, the renal pathological damage in DKD showed varying degrees of improvement after intervention with SYFS formula and DA. Compared with control group, the DKD group showed significantly increased FPG, kidney weight to body weight ratio (kW/BW), Scr, and urine microalbumin to creatinine ratio (mA/uCr). After intervention with SYFS formula and DA, kW/BW, Scr, and mA/uCr all decreased to varying degrees (Figure 11B–E). Further validation of hub genes using whole transcriptome sequencing technology revealed that, except for SST which was not detected in the three groups and could not be statistically analyzed, the transcriptomic sequencing results of the other 4 hub genes are shown in Figure 11F–I. Compared to Control group, Mmp3, Mmp12, and Ptges were significantly increased in DKD group, and decreased after SYFS formula intervention (*p* < 0.05), while Dusp1 was significantly decreased in DKD group, and increased after SYFS formula intervention (*p* < 0.05).

Figure 11Transcriptomic validation: (A) HE, Masson, and PAS staining of control, DKD, SYFS, and DA groups; (B) FPG levels; (C) kW/BW levels; (D) Scr levels; (E) mA/uCr levels; (F) Mmp3 expression levels; (G) Mmp12 expression levels; (H) Ptges expression levels; (I) Dusp1 expression levels (compared with control group,  ^∗^ *p* < 0.05, ^∗∗^ *p* < 0.01; compared with DKD group,^#^ *p* < 0.05,^##^ *p* < 0.01).

**Figure:**
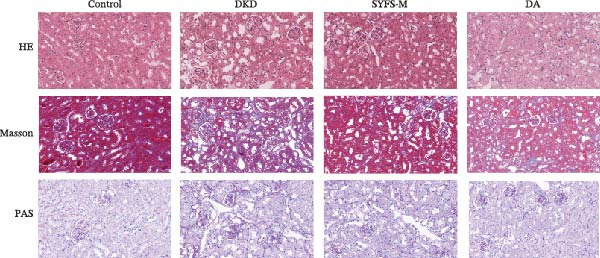
(A)

**Figure:**
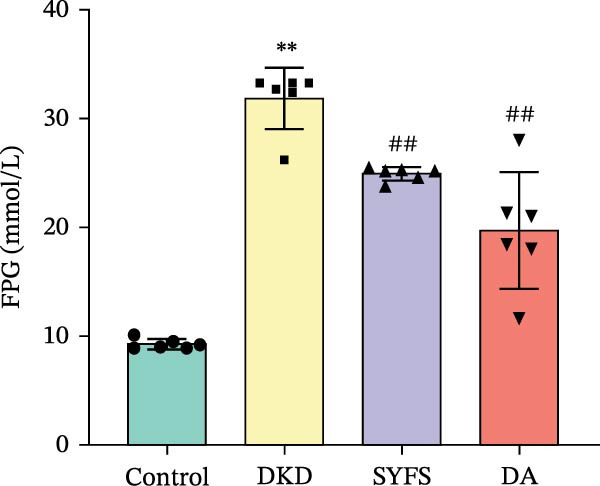
(B)

**Figure:**
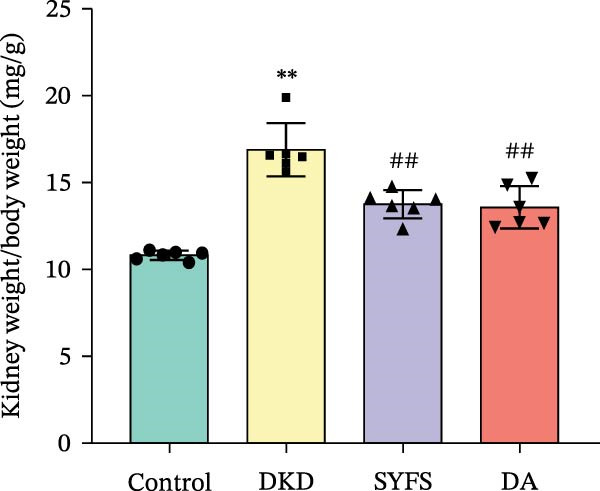
(C)

**Figure:**
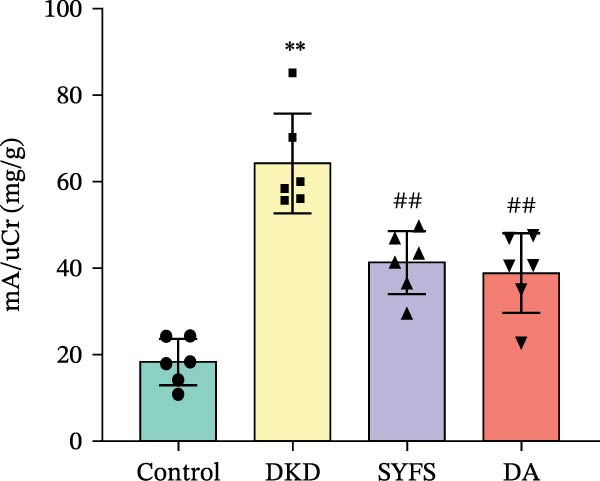
(D)

**Figure:**
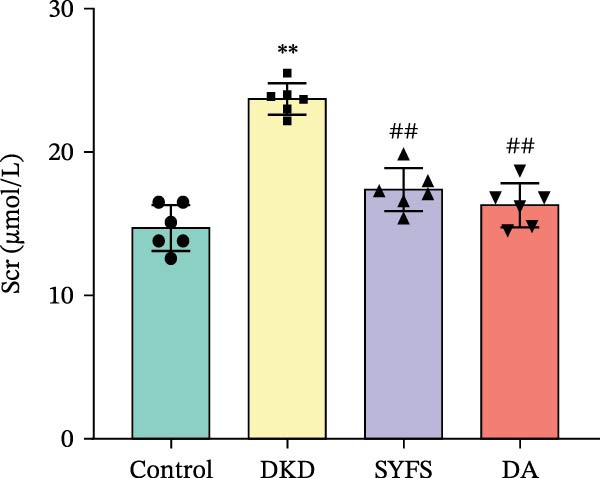
(E)

**Figure:**
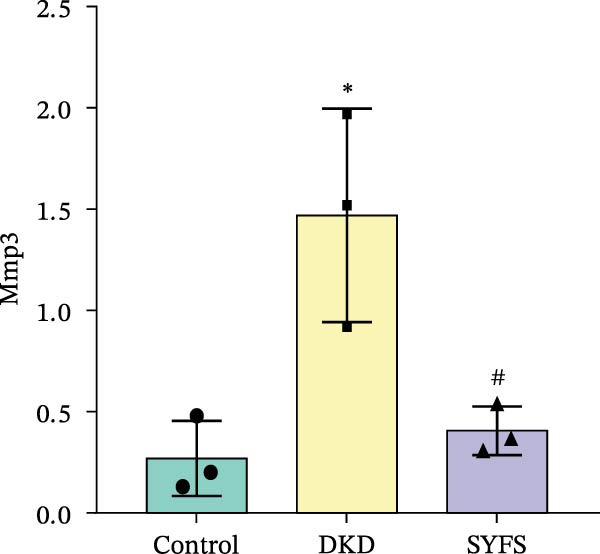
(F)

**Figure:**
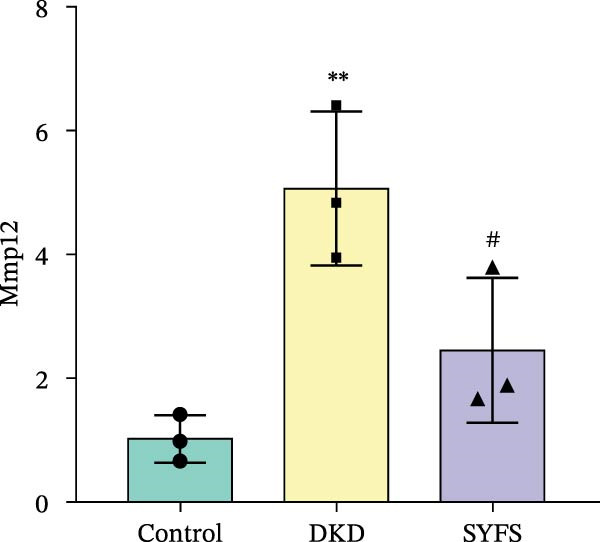
(G)

**Figure:**
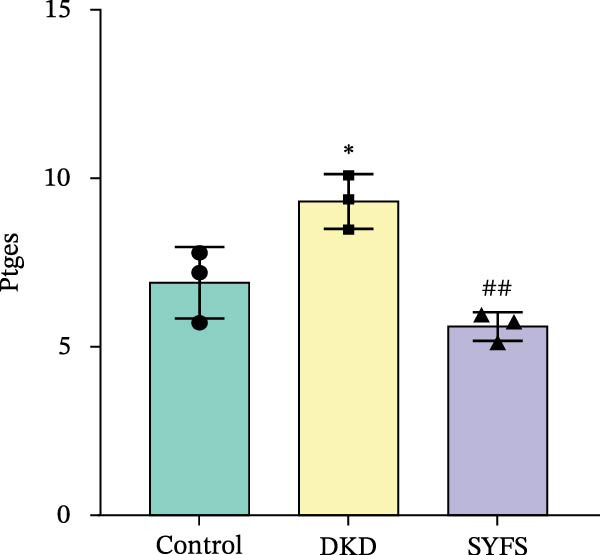
(H)

**Figure:**
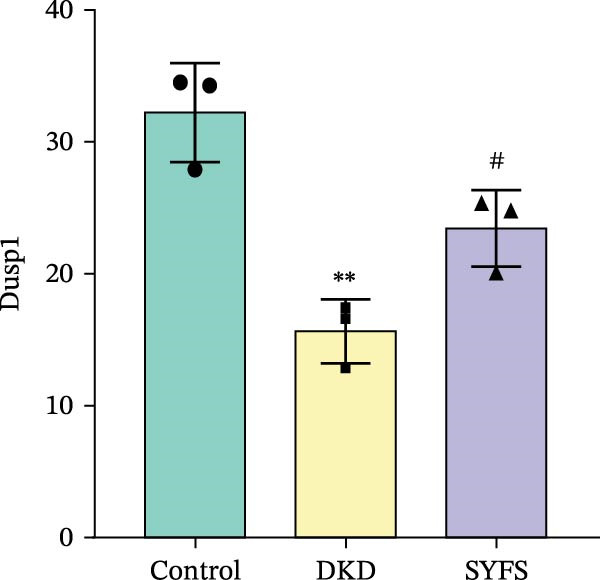
(I)

### 3.11. RT‐qPCR Analysis

RT‐qPCR analysis was performed on renal tissues from the Control, DKD, and SYFS groups. The results showed that, compared to the Control group, the expression of MMP3, MMP12, and PTGES in the DKD group was elevated, while the expression of DUSP1 and SST was reduced (*p* < 0.05). After SYFS treatment, the expression of MMP3, MMP12, and PTGES decreased, while the expression of DUSP1 increased (*p* < 0.05) (Figure 12A,B). The optimal treatment concentration and time of SYFS for HK‐2 cells were determined through CCK‐8 assay. After 24 h of SYFS treatment, no cytotoxicity was observed in HK‐2 cells at concentrations of 800 μg/mL or below; however, when the concentration was increased to 1600 μg/mL, cell viability decreased significantly. Therefore, concentrations of 800 μg/mL or below were selected as the safe concentration range for subsequent experiments (Figure 12C). DKD model was established by treating HK‐2 cells with 30 mmol/L glucose and 300 µmol/L PA. Compared to the Control group, the cell viability in the DKD group was significantly lower (*p* < 0.01). Compared to the model group, the cell viability in the SYFS treatment group at concentrations of 50–250 μg/mL significantly increased (*p* < 0.01), with a peak observed at 100 μg/mL, which resulted in the highest cell viability (Figure 12D). Therefore, 100 µg/mL was selected as the optimal treatment concentration of SYFS for subsequent experiments. The results showed that, compared to the Control group, the expression of MMP3, MMP12, and PTGES was elevated in the DKD group, while the expression of DUSP1 and SST was reduced (*p* < 0.05). After SYFS intervention, the expression of MMP3, MMP12, and PTGES decreased, while the expression of DUSP1 and SST increased (*p* < 0.05) (Figure 12E,F).

Figure 12RT‐qPCR analysis: (A–B) RT‐qPCR results from mouse kidney tissues; (C) effect of different concentrations of SYFS on the viability of HK‐2 cells; (D) effect of different concentrations of SYFS on cell viability in the DKD model; (E–F) RT‐qPCR results from HK‐2 cells (compared with control group,  ^∗^ *p* < 0.05, ^∗∗^ *p* < 0.01; compared with DKD group,^#^ *p* < 0.05,^##^ *p* < 0.01).

**Figure:**
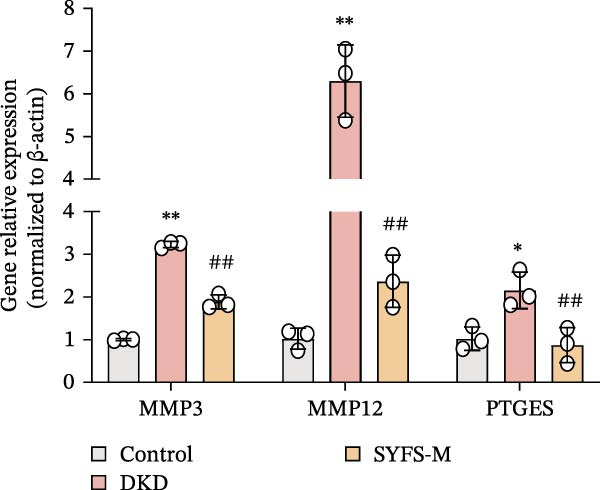
(A)

**Figure:**
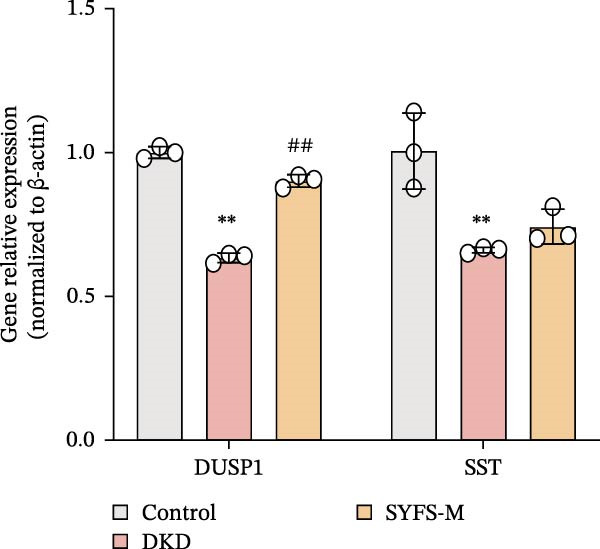
(B)

**Figure:**
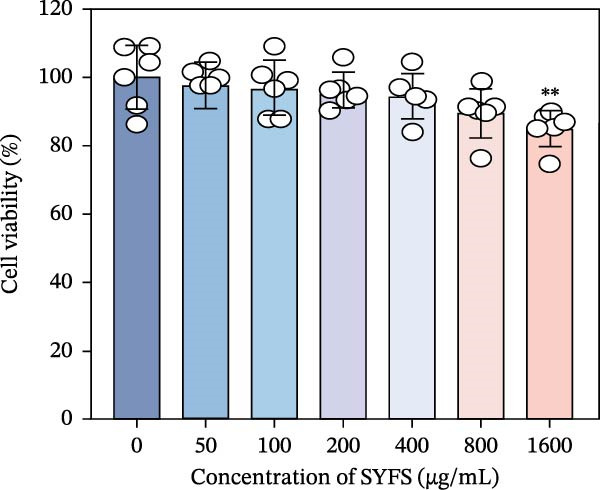
(C)

**Figure:**
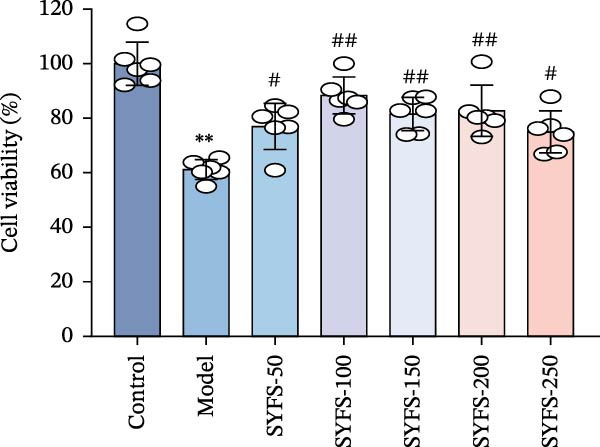
(D)

**Figure:**
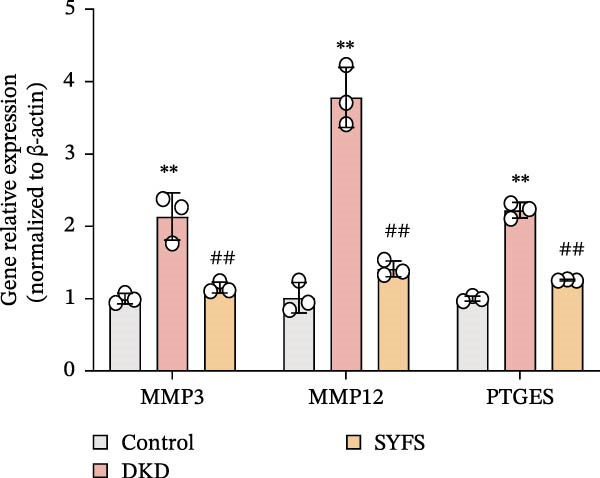
(E)

**Figure:**
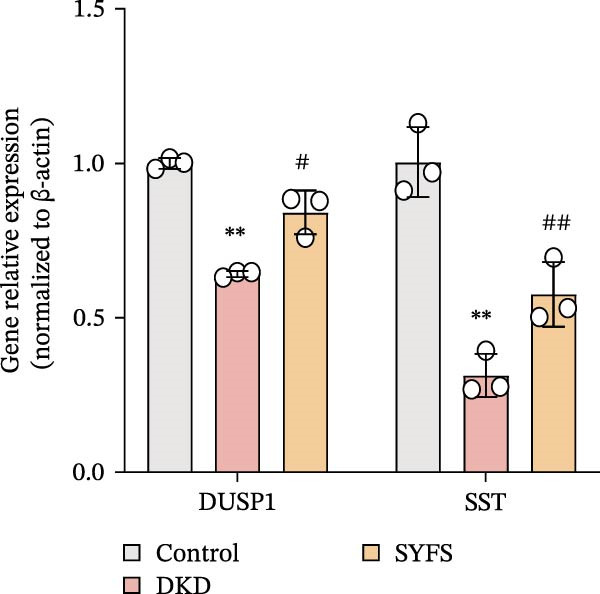
(F)

## 4. Discussion

DKD is a major cause of end‐stage renal disease, highlighting the urgent need to explore effective methods to halt its progression [27]. TCM has a long history in treating DKD, and meta‐analysis results indicate that the combination of Chinese and Western medicine for treating DKD shows significant efficacy in reducing 24 h proteinuria levels, improving renal function indicators, and optimizing lipid profiles [28, 29]. Identifying the therapeutic targets of TCM for diseases helps clarify their mechanisms of action, drives new drug development, promotes the modernization and internationalization of TCM, and optimizes clinical treatment strategies. Compared to most single‐method network pharmacology prediction studies, this work lies in the integration of high‐precision mass spectrometry identification, cross‐validation using eight ML algorithms, molecular docking and dynamics simulations, as well as in vivo experimental validation. A complete evidence chain was established, extending from computational predictions to biological function validation.

### 4.1. Mechanism of Synergistic Action of SYFS Formula’s Multiple Components

In this study, 154 ingredients were identified from the SYFS formula, including flavonoids, terpenoids, alkaloids, and their derivatives, which align with the “multi‐target treatment” theory of Chinese medicine. Among these, Naringenin chalcone, Palmatine, Oleanonic acid, *β*‐Elemonic acid, and Naringenin are the core components. Research has shown that Naringenin chalcone participates in inducing apoptosis and autophagy and regulates the PI3K/Akt signaling pathway [30]. It inhibits chronic inflammation in adipose tissue related to obesity, thus improving insulin resistance associated with obesity [31, 32]. Palmatine has various biological functions, such as regulating blood lipids, antibacterial, and anti‐inflammatory activities [33]. It reduces β‐cell apoptosis, increases β‐cell mass, and improves insulin secretion and alleviates insulin resistance by inhibiting the ERK and JNK signaling pathways, ultimately improving glucose tolerance induced by an HFD [34]. It also activates insulin release via the PI3K‐GLUT4 pathway [35], regulates NF‐κB/MAPK and Akt pathways to suppress inflammation and apoptosis, and prevents cisplatin‐induced acute kidney injury [36]. Oleanonic acid can inhibit carbohydrate‐metabolizing enzymes, promote glucose uptake, and improve blood lipid parameters, showing significant hypoglycemic and hypolipidemic activities [37]. *β*‐Elemonic acid induces apoptosis through inhibiting the Wnt/β‐catenin signaling pathway, endoplasmic reticulum stress‐mediated apoptosis, and activation of mitochondrial apoptosis pathways [38]. Naringenin reduces glomerular mesangial cell proliferation and ECM deposition by inhibiting the TGF‐β1/Smad3 and NF‐κB signaling pathways, thus alleviating renal fibrosis [39]. It also exerts significant therapeutic effects on DKD through multiple mechanisms, such as regulating endoplasmic reticulum stress, antioxidant stress, and antiapoptosis [40, 41]. These findings suggest that SYFS works through the synergistic action of multiple components targeting key pathological processes in DKD, including glucose and lipid metabolism, inflammation, oxidative stress, and cell apoptosis.

### 4.2. Biological Significance and Clinical Value of Hub Genes

The hub genes (MMP3, MMP12, PTGES, SST, DUSP1) selected through eight machine learning methods exhibit significant expression differences in DKD. MMPs (Matrix Metalloproteinases) are a class of zinc‐dependent proteinases that regulate various proteins, such as cell surface receptors, chemokines, and cytokines, by cleaving them. This regulation impacts cell signaling, migration, apoptosis, immune cell recruitment, and inflammation [42]. MMP3, for example, degrades the ECM and regulates the expression of inflammatory factors, promoting the infiltration of inflammatory cells [43]. MMP3 can degrade type IV collagen in the glomerular basement membrane, disrupting its integrity, which increases protein filtration. Plasma MMP3 levels are correlated with eGFR and plasma creatinine levels, making it a potential biomarker for early kidney damage [44]. MMP12, produced by infiltrating macrophages, plays an important role in inflammation, cell proliferation, and tissue remodeling [45]. The absence of MMP‐12 significantly reduces macrophage infiltration in the glomeruli, alleviating the inflammatory response [46]. Plasma proteomic results suggest that MMP‐12 may serve as a cardiovascular risk marker in CKD patients [47]. PTGES catalyzes the synthesis of prostaglandin E2 (PGE2) [48]. In DKD, the increased activity of mPGES‐1 leads to the excessive production of PGE2, which affects GFR and renal blood flow, exacerbating inflammation and oxidative stress, and damaging the glomeruli and renal tubules [49]. SST regulates β‐cell function by acting on the SSTR3 receptor on primary cilia in the islets, reducing cAMP levels and triggering Ca^2+^signaling. It also regulates GLI2 nuclear translocation and gene expression [50]. In DKD, SST reduces glomerular hypertrophy and proteinuria by inhibiting the IGF‐1 and GH/IGF‐1 axis, thereby improving kidney function [51, 52]. DUSP1, a negative regulator of the MAPK pathway, alleviates high glucose‐induced tubular cell injury by restoring autophagic flux, improving mitochondrial function, reducing ROS production, and preventing apoptosis [52, 53]. It also exerts antifibrotic effects by inhibiting the p38MAPK and ERK1/2 signaling pathways, thus reducing the expression of ECM components [54]. The Nomogram model built based on these genes (AUC = 0.963) demonstrates high diagnostic value and is significantly correlated with kidney function indicators (GFR). Immune infiltration analysis shows increased infiltration of M1 and M2 macrophages, regulatory T cells, and dendritic cells in the DKD group, suggesting that immune imbalance contributes to the progression of DKD, which is consistent with previous studies [3, 55]. Consensus clustering further classified DKD into two subtypes: high immune infiltration (C2) and low immune infiltration (C1). There is a complex correlation between hub genes and immune cells, providing theoretical support for the exploration of precision subtype‐based treatments.

### 4.3. Molecular Docking and Experiment Verification

The core active ingredients in the SYFS formula may regulate hub genes through multiple pathways. Previous studies have shown that the AMPK/mTOR [56], PI3K/Akt [57], NF‐κB [58, 59], and TGF‐β/Smad [60] signaling pathways are crucial regulatory pathways for MMP3, MMP12, PTGES, and other factors. As previously mentioned, the core active ingredients, Naringenin chalcone [30], Palmatine [36, 61], and Naringenin [39], may indirectly affect the expression of hub genes through the regulation of these mechanisms. Currently, using ML and network analysis to uncover the mechanisms of complex diseases, combined with molecular docking and dynamics simulations to accelerate the screening of bioactive natural compounds, has become a new trend [62–64]. Further molecular docking studies showed that the components of the SYFS formula had a strong binding affinity with the target. Naringenin and MMP3 having the most stable binding energy (−9.5 kcal/mol), and MD simulation further confirmed its stability. Transcriptomic and RT‐qPCR results from animal experiments showed that pathological damage, including glomerular hypertrophy and fibrosis, was significantly improved. After treatment with SYFS, compared to the DKD group, the expression of MMP3, MMP12, and PTGES decreased, while the expression of DUSP1 increased. The consistency between the experimental data and bioinformatics predictions validates the effectiveness of our integrated computational and experimental approach.

### 4.4. Limitations and Future Prospects

Although this study systematically revealed the action network of SYFS formula, there are still limitations: (1) the animal model does not fully replicate the chronic progression characteristics of human DKD; (2) the study failed to perform absolute quantification of the key components of the SYFS formula; and (3) the bioactive components predicted by network pharmacology lack verification regarding in vivo bioavailability and pharmacologically relevant concentrations. This limitation causes the true active substance basis of the SYFS formula to remain at a speculative stage. Future studies should experimentally validate the predicted signaling pathways, such as the MAPK pathway, in order to confirm the mechanisms of action of the SYFS formulation as indicated by bioinformatics analyses. Additionally, subsequent research should perform absolute quantification of the key components in the decoction and conduct pharmacokinetic studies. These investigations will help distinguish the “driving” components that exert direct effects from the “promoting” components that contribute synergistically.

## 5. Conclusion

This study systematically uncovers the potential mechanisms of the SYFS formula in treating DKD by integrating network pharmacology, bioinformatics analysis, and experimental validation. The study found that SYFS formula exerts therapeutic effects by synergistically acting through multiple ingredients (such as flavonoids, terpenes, and alkaloids) on key targets (such as MMP3, MMP12, PTGES, SST, and DUSP1), regulating inflammatory responses, cell chemotaxis, and the immune microenvironment. These findings decode the overall regulatory network of SYFS formula, linking TCM theories with modern mechanistic insights.

## Author Contributions

**Yi Kang**: conceptualization, methodology, data curation, formal analysis, writing – original draft, writing – review and editing. **Qian Jin**: conceptualization, methodology, data curation, formal analysis, writing – original draft, writing – review and editing. **Mengqi Zhou**: formal analysis, data curation, visualization, writing – original draft. **Huijuan Zheng**: data curation, formal analysis, validation, writing – original draft. **Danwen Li**: methodology, investigation, data analysis, writing – original draft. **Xuezhe Wang**: data curation, investigation, software analysis, writing – original draft. **Jingwei Zhou**: methodology, investigation, writing – original draft, writing – review and editing. **Jie Lv**: conceptualization, supervision, project administration, writing – review and editing, funding acquisition. **Yaoxian Wang**: conceptualization, supervision, project administration, writing – review and editing, funding acquisition.

## Funding

This research was funded by Traditional Chinese Medicine Scientific Research Project of Hebei Provincial Administration of Traditional Chinese Medicine (Grant B2026022 to Jie Lv), Clinical Research and Achievement Transformation Capability Enhancement Pilot Project of Dongzhimen Hospital, Beijing University of Chinese Medicine (Grant DZMG‐ZLZX‐25022 to Jie Lv), and the National Natural Science Foundation of China (Grant 82174342 to Yaoxian Wang).

## Disclosure

All authors read and approved the final manuscript.

## Ethics Statement

All experimental procedures were approved by the Ethics Committee of Beijing University of Chinese Medicine (BUCM‐2023120104−4282).

## Conflicts of Interest

The authors declare no conflicts of interest.

## Supporting Information

Additional supporting information can be found online in the Supporting Information section.

## Supporting information


**Supporting Information 1** 1: UHPLC‐MS/MS analysis. 2: Animal experiment verification. 3: Molecular dynamics simulation. 4: Supporting Figures.

**Supporting Information 2** Table S1 Overview of the datasets. Table S2 Pharmaceutical ingredient. Table S3 Drug targets. Table S4 Differentially expressed genes. Table S5 WGCNA. Table S6 Overlapping genes.

## Data Availability

Data available upon request from the authors.
