# Supplementary material for: Compound Analysis and Mechanistic Exploration of Shen‐Yan‐Fang‐Shuai Formula in Diabetic Kidney Disease: An UHPLC‐MS/MS and Bioinformatics Study
Source: J Diabetes Res. 2026 Apr 24;2026:7530512. doi: 10.1155/jdr/7530512 (PMC13109625; doi:10.1155/jdr/7530512)
Supplement: Supplementary file 1 — Supporting Information 1 1: UHPLC‐MS/MS analysis. 2: Animal experiment verification. 3: Molecular dynamics simulation. 4: Supporting Figures. [file JDR-2026-7530512-s002.docx]

**Supplementary Material**

**1 UHPLC-MS/MS analysis**

Chromatographic separation was performed on an ACQUITY BEH C18 column (100 mm × 2.1 mm id, 1.7 μm; Waters, USA). The mobile phase consisted of 0.1% formic acid in water:acetonitrile (2:98, v/v) (solvent A) and 0.1% formic acid in water:acetonitrile (solvent B), with a flow rate of 0.40 mL/min at a column temperature of 40°C. The injection volume was 3 μL. Mass spectrometry conditions: The UPLC system was coupled to a Thermo UHPLC-Q high-resolution mass spectrometer equipped with an electrospray ionization (ESI) source, operating in both positive and negative modes. Optimal conditions were set as follows: source temperature 450°C; sheath gas flow rate 50 arb; auxiliary gas flow rate 13 arb; ion spray floating voltage (ISVF) at -3000V and +3500V respectively; normalized collision energy with rolling MS/MS at 20-40-60V. The full MS resolution was 70,000 and MS/MS resolution was 17,500. UHPLC-MS raw data were processed using Progenesis QI software (Waters, Milford, USA) for baseline filtering, peak identification, integration, retention time correction, and peak alignment, converting raw data into a universal format. The resulting data matrix containing sample names, m/z, retention times, and peak intensities was exported for further analysis.

**Chromatographic elution gradient condition**

| Time（min） | %A | %B |
| --- | --- | --- |
| 0 | 98 | 2 |
| 0.5 | 98 | 2 |
| 3.5 | 75 | 25 |
| 7.5 | 65 | 35 |
| 11 | 50 | 50 |
| 13 | 5 | 95 |
| 14.4 | 5 | 95 |
| 14.5 | 98 | 2 |
| 16 | 98 | 2 |

**2 Animal experiment verification**

Forty-eight SPF-grade male C57BL/6J mice were housed in the SPF-grade animal facility at Beijing University of Chinese Medicine under controlled conditions of 24 ± 2°C, relative humidity of 40%-70%, and a 12-hour light/dark cycle. A high-fat diet combined with streptozotocin (STZ) was used to induce a DKD mouse model. The control group was fed a standard diet, while the model group was given a 60% high-fat diet. After 4 weeks, the model group was intraperitoneally injected with STZ (50 mg/kg, dissolved in 0.1 mol/L pH 4.5 citrate buffer) daily for 5 consecutive days, while the control group received an equal volume of buffer solution. The success of the model was confirmed by the criteria of three consecutive fasting blood glucose measurements ≥ 16.7 mmol/L.

**3 Molecular dynamics simulation**

The molecular dynamics (MD) simulations of the protein-ligand complex were carried out using the GROMACS 2025.2 package. The system was placed in a periodic boundary cubic box, solvated with the CHARMM36 force field and the TIP3P water model, with ions added to a physiological concentration (150 mM NaCl). After energy minimization and a stepwise NVT/NPT equilibration (each for 100 ps) under positional restraints, a 100 ns production run was conducted with an integration timestep of 2 fs. The trajectory was processed to remove periodic artifacts and the system was centered. The analysis included the root-mean-square deviation (RMSD) of the protein backbone Cα atoms, the radius of gyration (Rg), root-mean-square fluctuation (RMSF) of residues, solvent-accessible surface area (SASA), and the number of hydrogen bonds between the protein and ligand.

In this study, the molecular mechanics Poisson-Boltzmann surface area (MM-PBSA) method was employed to calculate the binding free energy (ΔGbind), an efficient computational framework suitable for assessing the binding affinity of protein-ligand complexes. This method quantifies the total binding free energy through post-processing of the molecular dynamics simulation trajectory and decomposes it into key components: molecular mechanics interaction energy (ΔEMM), including van der Waals energy (ΔEvdW) and electrostatic interaction energy (ΔEele), as well as solvation free energy—polar solvation energy (ΔGPB) and non-polar solvation energy (ΔGSA). While MM-PBSA is less accurate than free energy perturbation (FEP) or thermodynamic integration (TI) methods, it requires less computational cost and is suitable for distinguishing binding strength and performing molecular recognition analysis in large-scale systems. To further explore the binding mechanism, residue-level free energy decomposition analysis was performed. This method quantitatively assigns the total binding free energy to each amino acid residue of the protein receptor, calculating the changes in van der Waals energy, electrostatic interaction energy, and solvation energy for each residue-ligand pair. The analysis identifies key "hotspot" residues within the binding pocket, providing molecular-level insights into the binding specificity and structural optimization.

Additionally, molecular electrostatic potential (ESP) analysis was conducted to visualize the charge distribution and illustrate the electrostatic environment of the molecule. Furthermore, we constructed two-dimensional (2D) and three-dimensional (3D) free energy landscapes (FEL) to visualize the conformational stability of the complex. These landscapes use the radius of gyration (Rg) and root-mean-square deviation (RMSD) as coordinates to display the free energy distribution of the system in conformational space. Deep blue low-energy regions indicate the global free energy minimum, corresponding to the most thermodynamically stable conformation.

**4 Supplementary Figures**


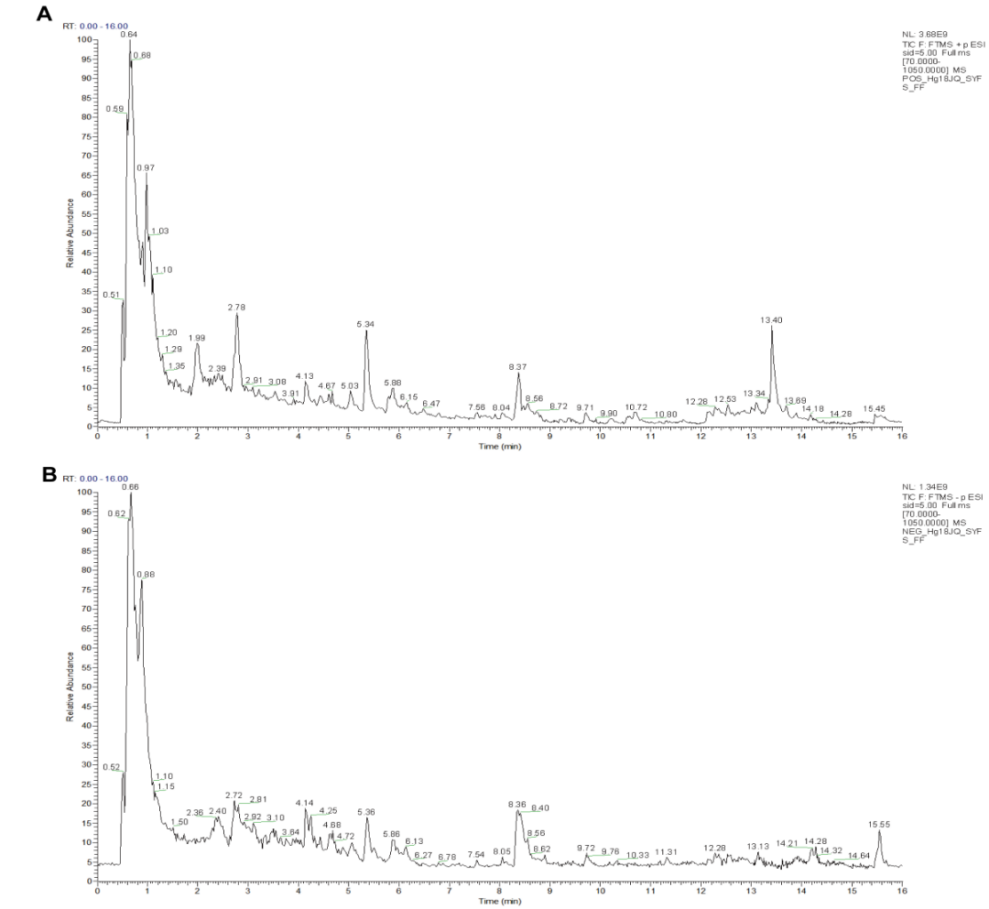


**Figure S1 Total ion flow map of SYFS formula in nephritis.** (A) Positive ion map. (B) Negative ion map

**.**
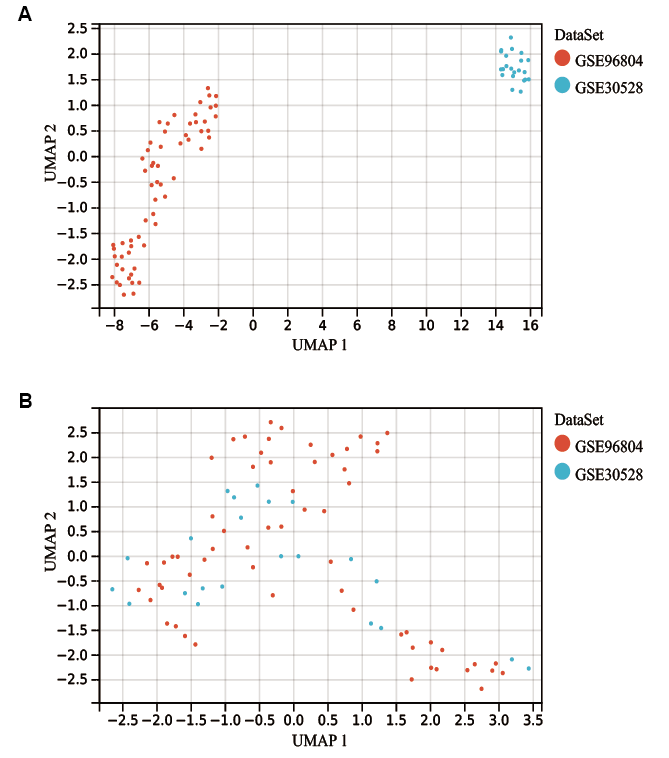


**Figure S2 Batch effect removal and data integration of GSE96804 and GSE30528 datasets.**

(A) Before batch effect removal: combined data showing the presence of batch effects.

(B) After batch effect removal: integrated dataset with batch effects successfully minimized.
